# Paracrine signal emanating from stressed cardiomyocytes aggravates inflammatory microenvironment in diabetic cardiomyopathy

**DOI:** 10.1016/j.isci.2022.103973

**Published:** 2022-02-23

**Authors:** Namrita Kaur, Andrea Ruiz-Velasco, Rida Raja, Gareth Howell, Jessica M. Miller, Riham R.E. Abouleisa, Qinghui Ou, Kimberly Mace, Susanne S. Hille, Norbert Frey, Pablo Binder, Craig P. Smith, Helene Fachim, Handrean Soran, Eileithyia Swanton, Tamer M.A. Mohamed, Oliver J. Müller, Xin Wang, Jonathan Chernoff, Elizabeth J. Cartwright, Wei Liu

**Affiliations:** 1 Faculty of Biology, Medicine, and Health, University of Manchester, Oxford Road, M13 9PT Manchester, UK; 2 Institute of Molecular Cardiology, University of Louisville, 580 S Preston Street, Louisville, KY 40202, USA; 3 Department of Internal Medicine III, University of Kiel, Kiel, Germany; 4 DZHK, German Centre for Cardiovascular Research, Partner Site Hamburg/Kiel/Lübeck, Kiel, Germany; 5 Department of Cardiology, Angiology and Pneumology, University of Heidelberg, Heidelberg, Germany; 6 Cancer Biology Program, Fox Chase Cancer Center, 333 Cottman Avenue, Philadelphia, PA 19111, USA

**Keywords:** Cardiovascular medicine, Biological sciences, Immunology, Cell biology

## Abstract

Myocardial inflammation contributes to cardiomyopathy in diabetic patients through incompletely defined underlying mechanisms. In both human and time-course experimental samples, diabetic hearts exhibited abnormal ER, with a maladaptive shift over time in rodents. Furthermore, as a cardiac ER dysfunction model, mice with cardiac-specific p21-activated kinase 2 (PAK2) deletion exhibited heightened myocardial inflammatory response in diabetes. Mechanistically, maladaptive ER stress-induced CCAAT/enhancer-binding protein homologous protein (CHOP) is a novel transcriptional regulator of cardiac high-mobility group box-1 (HMGB1). Cardiac stress-induced release of HMGB1 facilitates M1 macrophage polarization, aggravating myocardial inflammation. Therapeutically, sequestering the extracellular HMGB1 using glycyrrhizin conferred cardioprotection through its anti-inflammatory action. Our findings also indicated that an intact cardiac ER function and protective effects of the antidiabetic drug interdependently attenuated the cardiac inflammation-induced dysfunction. Collectively, we introduce an ER stress-mediated cardiomyocyte-macrophage link, altering the macrophage response, thereby providing insight into therapeutic prospects for diabetes-associated cardiac dysfunction.

## Highlights

• In response to metabolic stress, loss of cardiac PAK2 leads to maladaptive ER stress
• Cardiac CHOP-upregulated HMGB1 promotes M1 macrophage polarization by paracrine action
• Pharmacological inhibition of HMGB1 decelerates myocardial inflammation
• Vildagliptin and remediated ER alleviate cardiac dysfunction in diabetes

## Graphical Abstract

**Figure:**
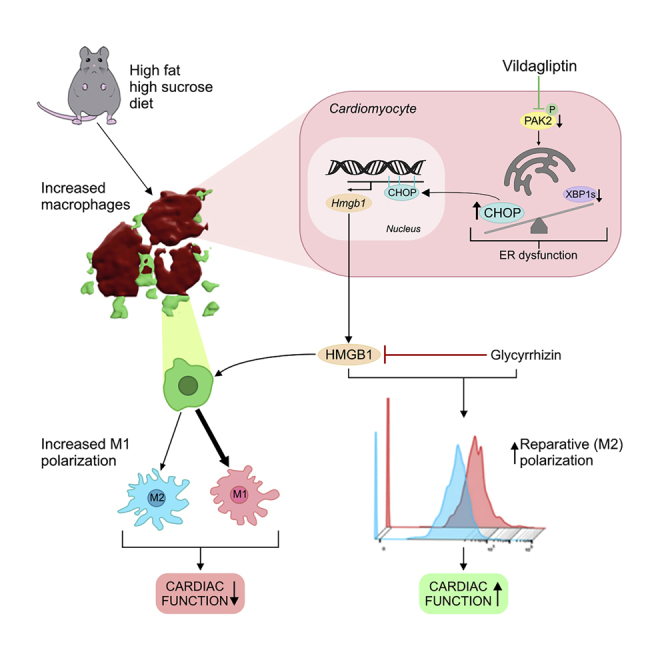

## Introduction

In clinical settings, heart failure (HF) is a serious comorbidity and a highly fatal condition in patients with type 2 diabetes (T2D), predicted to account for approximately 30% of hospital admissions with cardiovascular disease (CVD) (Shah et al., 2015). T2D is now recognized to have a detrimental impact on the myocardium leading to the onset and progression of cardiac dysfunction, termed diabetic cardiomyopathy (DCM) (Isfort et al., 2014). The underlying mechanism of DCM's etiopathogenesis involves a complex interplay of intra- and intercellular signaling (Frati et al., 2017). However, the integration of these signaling processes is incompletely defined.

The incidence of cardiac dysfunction in diabetes is at least partially attributable to uncontrolled myocardial inflammation. In the diabetic heart, the first typology of cardiac inflammation is subclinical, initiated by the secretion of inflammatory cytokines, chemokines, adhesion molecules, and danger-associated molecular patterns (DAMPs) from stressed cardiomyocytes, fibroblasts, and endothelial cells (Frati et al., 2017). These inflammatory factors stimulate monocytes and lymphocytes to exaggerate the cardiac inflammatory microenvironment and contribute to cardiac pathological remodeling and dysfunction (Frieler and Mortensen, 2015; Sanders-van Wijk et al., 2020; Tan et al., 2020). Nonetheless, a translational gap remains to produce effective DCM treatments based on identifying anti-inflammatory targets.

Generally, DCM is characterized by co-manifestation of chronic ER stress and sterile inflammation in clinics (Jia et al., 2018) and pre-clinical studies (Tan et al., 2020; Wold et al., 2005). Mounting data imply that ER dysfunction drives the inflammatory milieu contributing to disease pathogenesis (Gotoh et al., 2011; Zhang and Kaufman, 2008). The molecular underpinnings of ER stress-associated inflammation are documented in cells such as adipocytes (Maris et al., 2012; Suzuki et al., 2017), neurons (Wang et al., 2016), and endothelial cells (Gao et al., 2011; Gargalovic et al., 2006), yet how compromised cardiac ER stress response facilitates inflammation in the diabetic heart remains unknown. Pathological factors, such as metabolic stress, prompts the accumulation of misfolded/unfolded proteins and trigger an adaptive ER stress response, also known as the unfolded protein response (UPR), to lessen the protein overload (Cnop et al., 2012). Therefore, the regulated ER response is paramount for optimal cellular functions, particularly in irreplaceable cardiomyocytes (Ren et al., 2021). Conversely, the ER stress response becomes detrimental in the face of chronic stress, such as diabetes (Yang et al., 2015). The maladaptive ER stress response can mediate inflammatory transcriptional profiles by activating ER-associated transcription factors, including CCAAT/enhancer-binding protein homologous protein (CHOP) (Hummasti and Hotamisligil, 2010). However, the exact mechanisms by which ER stress impacts myocardial inflammation in DCM progression remain unclear. We previously identified p21-activated kinase 2 (PAK2) as a modulator of the cardiac ER stress response. It plays a protective role under pressure overload stress by decelerating the transition from adaptive to detrimental ER stress response (Binder et al., 2019). However, whether the role of cardiac PAK2 in modulating ER stress is recapitulated in diabetes is unknown.

We aimed to investigate the molecular link between these concurrent pathological insults, ER stress and inflammation, in the diabetic heart. Over time, experimental DCM revealed that the loss of PAK2 disrupts ER homeostasis aiding heightened myocardial inflammation and dysfunction. Mechanistically, we identified that CHOP induces direct transcriptional upregulation of high-mobility group box-1 (HMGB1), and its active release from stressed cardiomyocytes drives paracrine M1 macrophage subsets, causing an adverse inflammatory reaction in myocardium. Therapeutically, activated-PAK2 remediated ER function, and extracellular inhibition of HMGB1 represses ER stress-induced inflammation and subsequent dysfunction in diabetes. In summary, our findings establish the dysregulated ER response etiology for adverse myocardial inflammation via HMGB1, also supporting treatment strategies for preventing DCM by reducing the pro-inflammatory paracrine signal from cardiomyocytes.

## Results

### Maladaptive cardiac ER stress response in DCM

**Figure 1:**
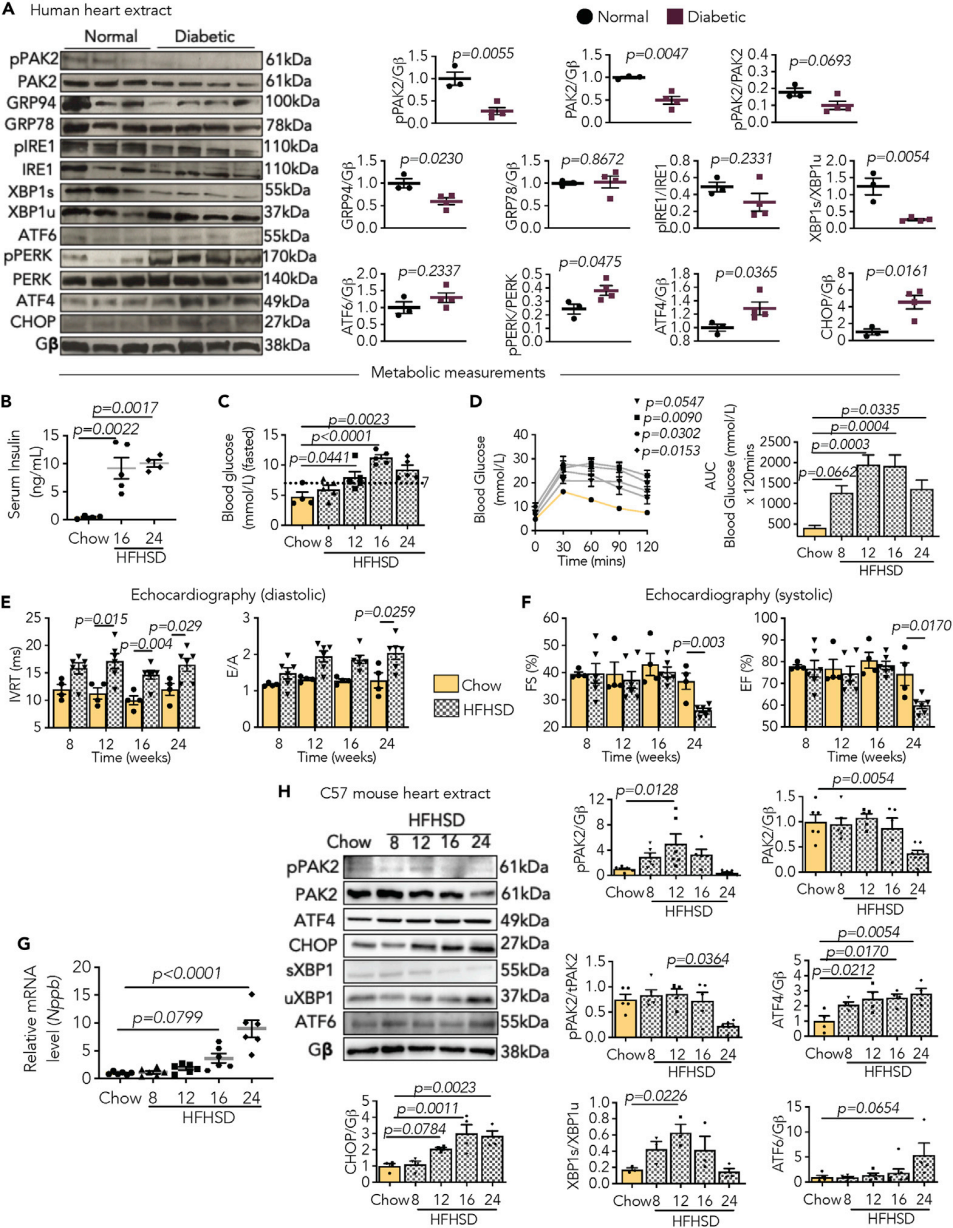
Time-course analyses exhibit cardiac dysfunction associated with maladaptive ER stress response following high-fat high-sucrose diet (HFHSD) (A) Representative images and quantification graphs (protein/Gβ ratio, AU) from densitometry analysis of PAK2 expression and phosphorylation (pPAK2) and ER stress markers from heart lysates of human subjects. ER stress markers include GRP94, GRP78, phosphorylated IRE1, IRE1, spliced XBP1 (XBP1s), unspliced XBP1 (XBP1u), ATF6, phosphorylated PERK (pPERK), PERK, ATF4, and CHOP. (B–D) Data graphs showing (B) serum insulin, (C) fasted blood glucose, and (D) intraperitoneal glucose tolerance test after 8–24 weeks of feeding in C57BL/6J mice and bar graph displaying AUC. (E) Graphs depicting isovolumetric relaxation time (IVRT) and E/A mitral valve flow from pulse-wave Doppler imaging indicating diastolic function. (F) From M-mode echocardiography: fractional shortening (FS%) and ejection fraction (EF%) indicating systolic function. (G) Relative mRNA expression of *Nppb* (chow normalized to 1, AU, arbitrary unit). (H) Immunoblots and quantification graphs (protein/Gβ ratio, AU) from densitometry analysis of key ER stress transcription factors from heart lysates of C57BL/6J mice. Gβ is loading control. Each data point represents one animal or subject. N = 3–4/group in (A), N = 4–5/group in (B–D), N/group, chow = 4, HFHSD = 6 in (E and F), N = 6/group in (G), and N = 3–5/group in (H). All data are presented as mean ± SEM. *p* values (shown in each panel) versus corresponding controls determined by two-tailed Student's *t* test in (A); and by ANOVA with Tukey's/Sidak's post hoc tests in (B–H).

To assess ER stress response in DCM, we first examined the key pathways and chaperones required for ER function in the hearts of patients with diabetes. A decrease in the ratio of spliced X-box binding protein 1 (XBP1s) versus unspliced XBP1 (XBP1u) was observed in the diabetic human heart, whereas the protein kinase R (PKR)-like ER kinase (PERK) and activating transcription factor 4 (ATF4) branch was increased (Figure 1A). CHOP, mainly regulated by PERK-ATF4 and activating transcription factor 6 (ATF6), was significantly higher in the diabetic myocardium, although ATF6 remained unchanged (Figure 1A). Regarding the chaperones initiating the adaptive UPR, the GRP94 levels were reduced in the human heart with diabetes (Figure 1A). Noteworthy, under diabetes, the expression and phosphorylation of PAK2, an ER stress response modulator, were both decreased in the human heart (Figure 1A). The data elicit aberrant changes in ER stress response and its known modulator in the diabetic heart.

We then examined whether the onset and progression of DCM are associated with maladaptive cardiac ER stress in a pre-clinical model. For this, we fed 6-week-old male C57BL/6J mice with high-fat high-sucrose diet (HFHSD) for 8 to 24 weeks in a cross-sectional study. The mice displayed weight gain and higher serum cholesterol levels compared to age-matched chow-fed mice (Figures S1A and S1B). Diet-induced diabetes was verified by increased serum insulin, fasting blood glucose, and impaired glucose tolerance (Figures 1B–1D). As a result of sustained metabolic stress, cardiac dysfunction was exemplified by a prolonged isovolumetric relaxation time (IVRT), a rising E/A ratio (Figure 1E), and both reduced fractional shortening (FS%) and ejection fraction (EF%) (Figure 1F), albeit unaffected cardiac conductivity (Table S1). Increased left ventricular mass (LV mass), enlarged relative wall thickness (RWT), and end-diastolic posterior wall thickness (dPW) were also observed during diabetes (Figures S1C–S1E). The transition to cardiac dilation occurred after 24 weeks of HFHSD feeding, as indicated by reduced RWT, and enlarged left ventricular chamber (LVEsD) with an increase in *Nppb* levels (Figures S1F and 1G). The deleterious heart function was associated with cardiomyocyte hypertrophy and increased fibrosis in HFHSD-fed mice (Figures S1G and S1H). The key ER-associated transcription factors were examined to establish the dysregulated ER stress response in the myocardium from diabetic mice. Interestingly, XBP1s was activated at the early stages of diabetes but was downregulated later (Figure 1H). Cardiac ATF4 remained enriched after 12 weeks of HFHSD feeding, whereas ATF6 was increased at a later stage (Figure 1H). Moreover, CHOP expression increased gradually in the mouse myocardium after HFHSD feeding (Figure 1H). Consistent with the clinical findings, PAK2 activation was also enhanced in the myocardium during the early stages of diabetes, with a significant decline in expression and phosphorylation levels later (Figure 1H). Finally, swollen ER was observed in the failing diabetic mouse heart (Figure S2), suggesting a role for the maladaptive ER stress response in DCM development.

### Myocardial inflammation in the heart from diet-induced diabetes

**Figure 2:**
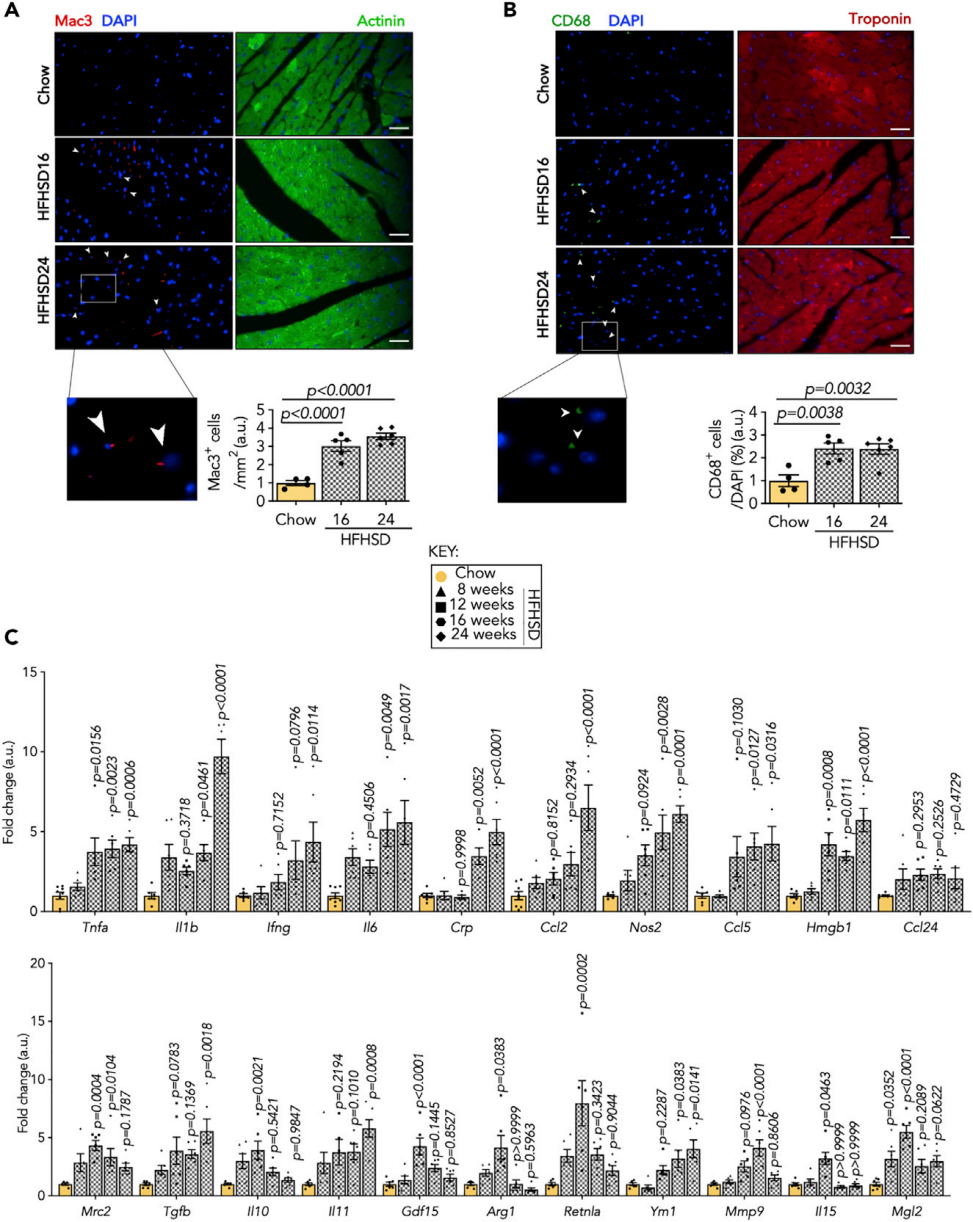
Perturbed ER homeostasis exacerbates myocardial inflammatory reaction in the diabetic myocardium (A and B) Representative immunofluorescence staining (scale bar: 25 μm) of (A) Mac3- (red) and B CD68- (green)-positive non-cardiomyocytes in heart sections of C57BL/6J mice fed with HFHSD indicating myocardial inflammation. Nuclei are stained with DAPI (blue). Cardiomyocytes are counterstained with α-actinin (green) in (A) and with troponin (red) in (B). (C) Relative mRNA expression (normalized to chow, AU) of pro- and anti-inflammatory markers from C57BL/6J hearts. *Inset: key for data points*; each data point represents one animal. N = 4–8/group. All data are presented as mean ± SEM. *p* values (shown in each panel) versus corresponding controls determined by ANOVA with Tukey's post hoc tests.

To investigate inflammation as a mechanism of DCM pathogenesis, the myocardial inflammatory response was assessed. Following 16 weeks of HFHSD feeding, we examined macrophages in the diabetic heart using immunofluorescence and found that Mac3 and CD68 were evident (Figures 2A and 2B). However, neutrophil (Ly6G) infiltration was not apparent in the diabetic myocardium (Figure S3). Cytokines facilitate immune cell activation and initiation of inflammatory responses. Hence, we tested the inflammatory profiles of the myocardium at different stages of diabetes using qPCR. The major pro-inflammatory cytokines, such as *Tnfa*, *Il1b*, *Ifng*, *Il6*, and C-reactive protein (*Crp*), were increased after 16 weeks of HFHSD feeding, reaching a peak at a later stage (Figure 2C). Interestingly, *Hmgb1* was elevated as early as 12 weeks post HFHSD (Figure 2C). In contrast, it was observed that the cytokines acting as anti-inflammatory factors, such as in *Il10*, *Il15*, *Mmp9*, and growth differentiation factor 15 (*Gdf15*) first peaked and eventually decreased in the diabetic mouse heart (Figure 2C), while *Tgfb* and *Il11* displayed a continuous and robust increase until 24 weeks (Figure 2C). Besides, upregulation of the chemokines triggering macrophages activation, such as monocyte chemoattractant protein-1 (*Mcp1/Ccl2*), *Ccl5*, and *Ccl24*, were present in the myocardium upon 16–24 weeks of HFHSD (Figure 2C), indicating that the initial rise in cytokine levels likely originates from the myocardium before macrophage appearance.

The macrophage-facilitated inflammatory response is composed of an elaborate equilibrium of pro- and anti-inflammatory signatures (Bajpai and Tilley, 2018). Furthermore, the prototypical markers distinguishing M1 and M2 subsets were measured to assess macrophage phenotypes in diabetic hearts. Notably, nitric oxide synthase 2 (*Nos2*), a key M1 subtype determinant, was upregulated in the mouse myocardium after 16 weeks of HFHSD feeding, with an even higher expression at 24 weeks (Figure 2C). Conversely, the genes characterizing M2 macrophages, including mannose receptor 2 (*Mrc2*), arginase 1 (*Arg1*), resistin-like alpha (*Retnla*), beta-N-acetylhexosaminidase (*Ym1*), and galactose N-acetyl-galactosamine-specific lectin 2 (*Mgl2*), were enriched in the mouse myocardium at 12 weeks of HFHSD feeding, but were reduced at the later stages (Figure 2C). Thus, we infer that the development of DCM is accompanied by notable inflammation with a predominance of M1 macrophages in the myocardium.

### Malfunctioned cardiac ER prompts myocardial inflammation in diabetes

As observed in heart, under the stimulation of high fatty acid and glucose (HFHG), an *in vitro* model displayed an oscillating pattern in PAK2 phosphorylation at different time points, coinciding with the transition from an adaptive UPR to a maladaptive ER stress response (Figures S4A and S4B). PAK2 activation was likely through both Cdc42 and Rac1, which are the putative activators of the PAKs family (Renkema et al., 2002), as its phosphorylation was diminished in both Cdc42- and Rac1-deficient cells (Figures S4C and S4D). Furthermore, we utilized *Pak2*-deficient and -overexpressing H9C2 cells with HFHG stimulation to assess whether PAK2 is required for ER housekeeping under diabetic conditions. *Pak2*-knockdown cells exhibited an aberrant ER function, which was determined by increased CHOP along with dampened XBP1s levels (Figure S5A). Conversely, the overexpression of PAK2 rescued XBP1s and suppressed CHOP under HFHG (Figure S5B). These observations support cardiac PAK2 regulation of ER stress pathways under diabetic stress.

**Figure 3:**
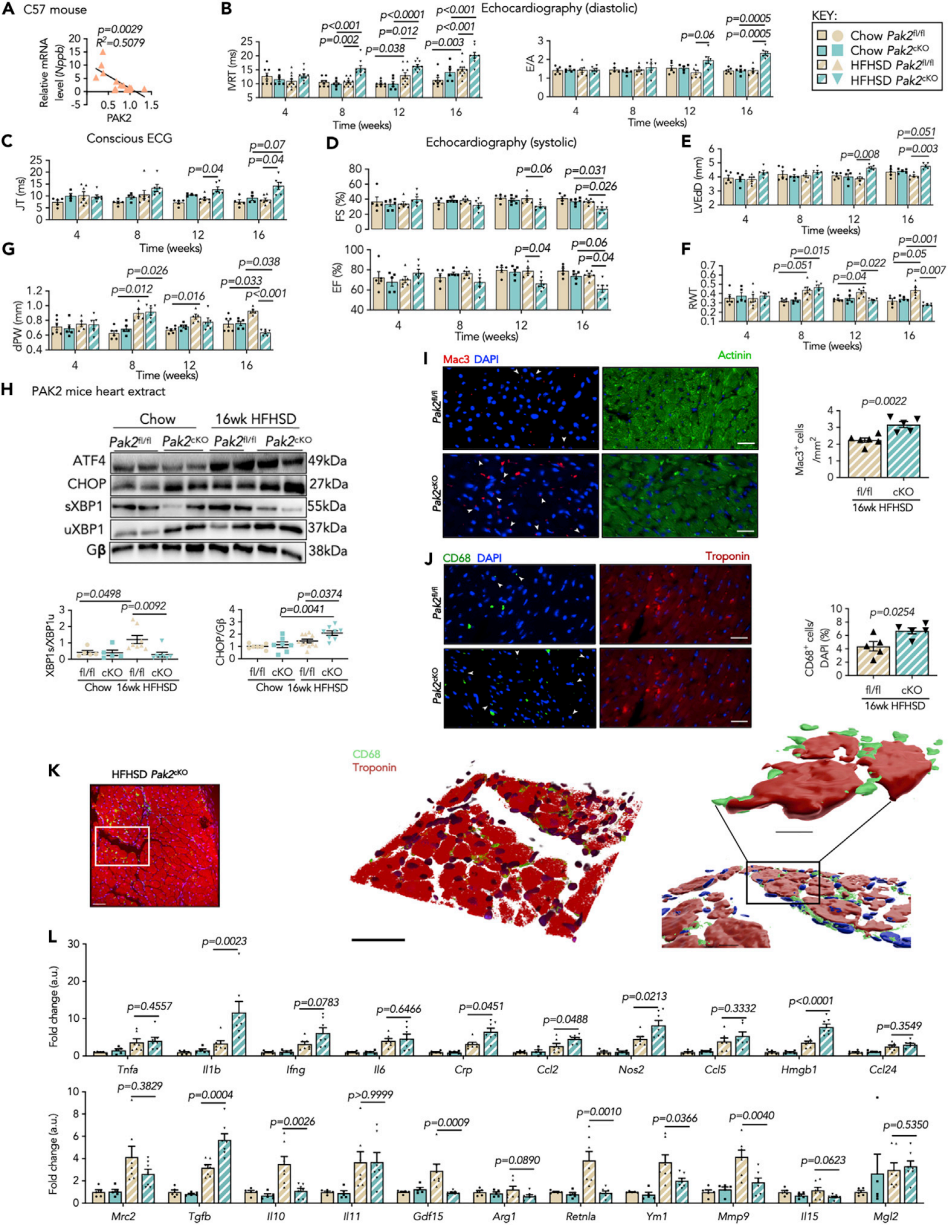
PAK2 deficiency-induced maladaptive cardiac ER stress-associated myocardial inflammation and cardiac dysfunction in diabetes (A) Correlation between *Nppb* transcript and PAK2 protein level in C57BL/6J mice hearts (N = 15 pairs). (B and C) Graphs depicting (B) IVRT and E/A mitral valve flow from pulse-wave Doppler imaging and (C) JT segment from ECG. (D–G) From M-mode echocardiography (D) calculated FS% and EF%, (E) LV-end diastolic diameter (LVEdD), (F) relative wall thickness (RWT), and (G) diastolic posterior wall (dPW) thickness*;* N = 5–9/group in (B–G). (H) Immunoblot images of ER stress markers from heart lysates of *Pak2*^*fl/fl*^ and *Pak2*^*cKO*^ mice. Images are representative of N = 5–8/group. Changes of XBP1s and CHOP (N = 5–11/group) displaying maladaptive ER stress response. Gβ is loading control. (I and J) Representative immunofluorescence staining (scale bar: 25 μm) of (I) Mac3- (red) and (J) CD68- (green)-positive non-cardiomyocytes in heart sections of *Pak2*^*fl/fl*^ and *Pak2*^*cKO*^ mice fed with HFHSD indicating myocardial inflammation. Nuclei are stained with DAPI (blue). Cardiomyocytes are counterstained with α-actinin (green) in (I) and with troponin (red) in (J); (N = 6/group). (K) Localization of CD68 macrophages (green) around cardiomyocytes (red) stained with troponin. Images are representatives of hearts from mice displaying fluorescence, blend mode, and 3D surface reconstruction from left to right (Scale bar: 20 mm). (L) Relative mRNA expression (normalized to chow, AU) of pro- and anti-inflammatory markers from *Pak2*^*fl/fl*^ and *Pak2*^*cKO*^ hearts (N = 5–7/group). *Inset: key for data points*; each data point represents one animal. All data are presented as mean ± SEM. *p* values (shown in each panel) versus corresponding controls (Chow) determined by two-tailed Student's *t* test in (I and J); and by ANOVA with Tukey's/Sidak's post hoc tests in (B–H and L); Pearson R coefficient analysis in (A).

A negative correlation of myocardial *Nppb* and PAK2 in diabetic mice (Figure 3A) led us to use mice with cardiac-specific PAK2 deletion (*Pak2*^*cKO*^) to gain functional evidence that PAK2 regulates ER stress and cardiac function. *Pak2*^*cKO*^ and the control littermates (*Pak2*^*fl/fl*^) were fed with HFHSD for 16 weeks. Their BW increased comparably (Figure S6A), and their metabolic profiles were examined by measuring serum cholesterol (Figure S6B), insulin (Figure S6C), glucose levels (Figure S6D), and glucose tolerance test (Figure S6E), which demonstrated a comparable diabetic condition in both HFHSD-fed groups. This indicated that cardiac PAK2 loss does not affect the diet-impaired systemic metabolic changes. Cardiac function was assessed by echocardiography and conscious ECG (Tables S2 and S3). Diastolic dysfunction occurred in *Pak2*^*cKO*^ at an early stage of diabetes, as reflected by prolonged IVRT and increased E/A ratio 8–12 weeks post HFHSD feeding (Figure 3B). JT intervals, indicating ventricular repolarization, were longer after 16 weeks of HFHSD feeding (Figure 3C and Table S3), also supporting compromised diastolic abnormality. In other respect, systolic dysfunction was observed in *Pak2*^*cKO*^ mice fed with HFHSD, which was evidenced by reduced FS% and EF% (Figure 3D). Additionally, *Pak2*^*cKO*^ hearts displayed ventricular dilation, as corroborated by increased end-diastolic left ventricular internal diameter (LVEdD), and reduced RWT and dPW thickness (Figures 3E–3G). Cardiac remodeling in *Pak2*^*cKO*^ hearts was evident, as indicated by increased myocardial *Nppb*, cardiomyocyte size, and fibrosis (Figures S7A–S7C). Furthermore, maladaptive ER stress response was exacerbated in the *Pak2*^*cKO*^ myocardium after feeding with HFHSD, determined by higher CHOP expression (Figure 3H). Collectively, these results further substantiate the apparent importance of cardiac PAK2 in regulating ER stress response under diabetic stress.

Cumulative evidence demonstrates a crosstalk between ER stress and inflammation; however, whether the cardiac ER stress response is a cause or a consequence of inflammation in the heart remains elusive. Therefore, we elucidated the connection between the maladaptive ER stress and myocardial inflammation. *Pak2*^*cKO*^ hearts displayed severe inflammation following 16 weeks of HFHSD feeding, exemplified by a greater number of Mac3-labelled and CD68-positive non-cardiomyocytes in the myocardium (Figures 3I, 3J, and S8A). Notably, the abundant localization of CD68 macrophages adjacent to cardiomyocytes suggested a possible paracrine communication (Figures 3K and S8B). More convincingly, the assessment of cytokines in *Pak2*^*fl/fl*^ and *Pak2*^*cKO*^ myocardium showed that *Il1b*, *Crp*, *Mcp1*, *Ccl24*, and *Hmgb1* were higher in *Pak2*^*cKO*^; on the contrary, *Il10*, *Mmp9*, and *Gdf15* were lower in *Pak2*^*cKO*^ post HFHSD (Figure 3L). In addition, increased *Nos2* expression indicated elevated M1 macrophages in *Pak2*^*cKO*^ hearts, while *Retnla* and *Ym1* were scarce (Figure 3L). As such, the above findings illuminate the previously overlooked effects of cardiac ER function on myocardial inflammation in metabolic disorder.

### Cardiac-sourced HMGB1 contributes to myocardial inflammation in diabetes

**Figure 4:**
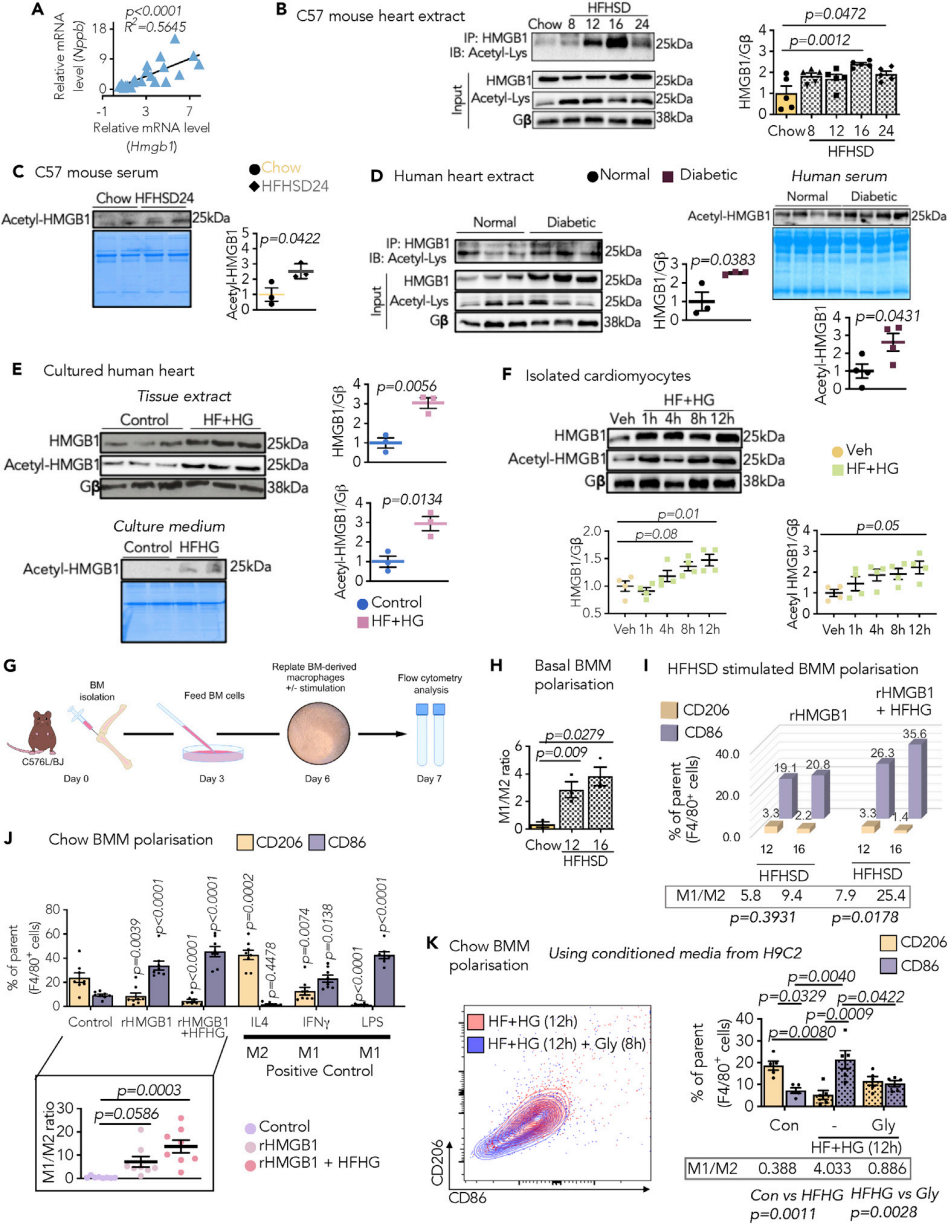
Cardiomyocyte-secreted HMGB1 participates in macrophage polarization toward M1 phenotype under diabetic condition (A) Correlation graph of relative *Hmgb1* expression to *Nppb* (N = 23 pairs) in C576L/BJ mouse hearts. (B and C) HMGB1 lysine acetylation from C57BL/6J mice (B) immunoprecipitation from hearts, and (C) serum immunoblot with quantification (N = 3–5/group, AU). (D) Immunoblots from hearts and serum from normal subjects and diabetic patients (N = 3–4/group, AU). (E and F) Immunoblot and quantification (E) tissue lysate and culture medium from cultured human hearts; (n = 3, technical repeat); and (F) isolated adult rat cardiomyocytes (ARCMs) (N = 4), stimulated with high fatty acid (500 μM) and high glucose (33 mM) (HFHG). (G) Experimental overview of macrophage differentiation from BM progenitors from C57BL/6J mice. The BM-derived macrophages (BMM) were selected as (Ly6G^−^F4/80^+^) and polarization markers include CD206 (M2) and CD86 (M1); gating in Figure S9A). (H) Data showing calculated M1/M2 ratio (normalized to chow, AU) of basal BMM from chow- and HFHSD-fed mice. (I–K) (I) 3D bar plot representing mean %CD206^+^ and %CD86^+^ macrophages from HFHSD-fed mice, stimulated with rHMGB1 (500 ng/mL) with and without HFHG (boxed, mean M1/M2 ratio, AU) (N = 3/group). %CD206^+^ and %CD86^+^ macrophages from chow-fed mice following (J) IL4 (20 ng/mL), IFNγ (50 ng/mL), lipopolysaccharide (LPS) (100 ng/mL), rHMGB1 (500 ng/mL) with and without HFHG stimulation, and (K) culture medium of HFHG stimulated H9C2 with and without glycyrrhizin (Gly). Boxed: Data showing mean M1/M2 ratio (AU) from each experiment (normalized to control, AU). All IP experiments (N = 1/group/cohort). Each data point represents one animal/experiment. All data are presented as mean ± SEM. *p* values (shown in each panel) versus corresponding controls determined by two-tailed Student's *t* test in (C–E) and by ANOVA with Tukey's/Sidak's post hoc tests in (B, F, and H–K). Pearson R coefficient analysis in (A).

Intracellular HMGB1 interacts with nucleosomes and transcription factors to facilitate gene regulation, whereas extracellular HMGB1 secreted from stressed cells, functions as a pro-inflammatory molecule (Chikhirzhina et al., 2020). Noticeably, myocardial *Hmgb1* was positively correlated with *Nppb*, supporting its detrimental effects on cardiac function (Figure 4A). In addition, lysine acetylation of HMGB1 is known to be vital for its active secretion from immune cells (Chikhirzhina et al., 2020). Of note, the elevated cardiac *Hmgb1* was observed in C57BL/6J mice as early as 12 weeks after HFHSD feeding (Figure 2C), its protein levels increased accordingly (Figure 4B). Interestingly, acetylated HMGB1 in the myocardium (Figure 4B) with concomitant increased HMGB1 levels in serum (Figure 4C) were observed in diabetic mice. More importantly, consistent observations of increased HMGB1 were made in the myocardium from human diabetic subjects with CVD and serum from patients with diabetes (Figure 4D). Further confirmation was provided by cultured human heart slices after HFHG stimulation (Ou et al., 2019), exhibiting more evident HMGB1 and its lysine-acetylated form following HFHG stress (Figure 4E), with a consequent increase in its secretion to the conditional medium (Figure 4E). Similar results were also observed in isolated rat cardiomyocytes following exposure to HFHG (Figure 4F). Taken together, these results reveal that hyperglycemia and hyperlipidemia induce cardiac-sourced HMGB1 upregulation and secretion in diabetes.

Prompted by the observations that an increased HMGB1 in the myocardium occurred prior to macrophage activation (Figures 2, 4B, and 4C), we speculated that HMGB1 actively secreted from stressed cardiomyocytes is crucial in shaping macrophage phenotype in diabetes. Hence, we explored whether and how cardiac-sourced HMGB1 exerts its pro-inflammatory action in the myocardium. Macrophages have the capability to directly recognize DAMPs and cytokines to provoke chronic inflammation in metabolic disorders (McNelis and Olefsky, 2014). We therefore obtained evidence of the interplay between cardiac HMGB1 and myocardial inflammatory reactions under diabetes. BM-derived macrophages (BMM) were isolated from mice fed with chow and HFHSD, following differentiation and stimulation, F4/80^+^ selected macrophages were classified as M1 and M2 subsets by CD86^+^ and CD206^+^ markers, respectively (Figures 4G and S9A). First, M1/M2 ratio increased following HFHSD feeding, indicating that the macrophage pro-inflammatory nature timed with increasing expression of myocardial HMGB1 (Figures 4H and S9B). Secondly, upon exposure to either recombinant HMGB1 (rHMGB1) or combination of rHMGB1 and HFHG, BMM isolated from mice at 12 and 16 weeks post HFHSD manifested significant polarization to M1 subtype (Figures 4I and S9C). Afterwards, we focused our efforts on the relevance of HMGB1 to the myocardial inflammatory response. Differentiated BMM from non-diabetic mice were treated with distinct stimulators of M1 and M2 macrophage subtypes. As expected, M1 macrophage activation by IFNγ was heightened in combination with lipopolysaccharides (LPS). Importantly, rHMGB1 regardless of HFHG also increased M1 polarization of IFNγ-primed macrophages (Figures 4J and S9D). In comparison to IL4-triggered M2 macrophage subtype, rHMGB1 impeded the M2 phenotype (Figures 4J and S9D). The H9C2-conditioned medium from long-term HFHG stimulation induced M1 polarization; however, glycyrrhizin reduced this polarization, when used as an adjuvant to sequester secreted HMGB1 (Figure 4K). Combined, these data suggest that the diabetic state instigates cardiac upregulation and secretion of HMGB1, further supporting the notion of HMGB1-driven macrophage polarization toward M1 phenotype.

### ER dysfunction-induced cardiac HMGB1 in diabetes

**Figure 5:**
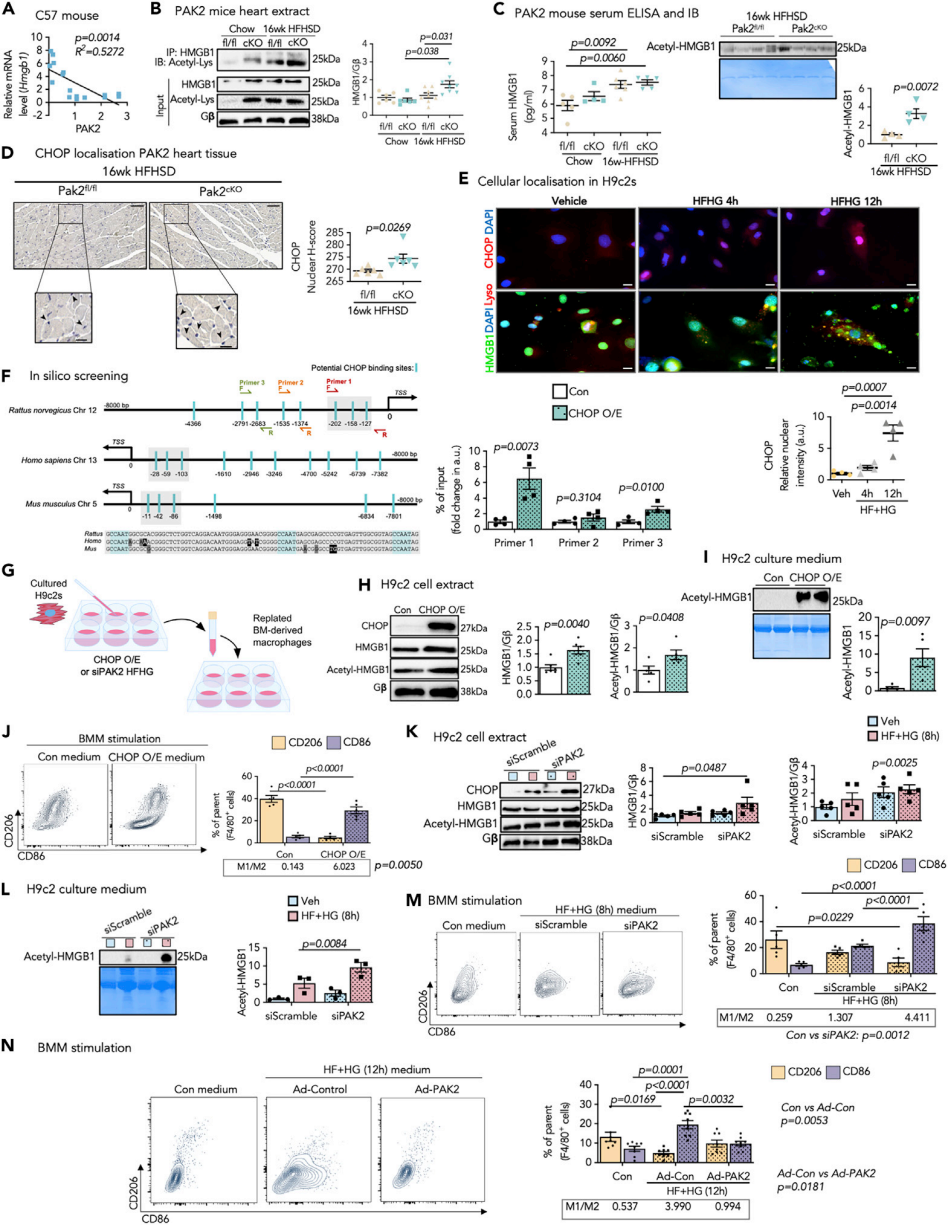
ER dysfunction facilitates cardiac HMGB1 expression and subsequent release (A) Correlation graph of relative *Hmgb1* expression to PAK2 (N = 16 pairs) expression in C576L/BJ hearts. (B-C) From *Pak2*^*fl/fl*^ and *Pak2*^*cKO*^ mice, (B) immunoprecipitation from hearts (N = 5–8/group, AU) for HMGB1 acetylation. (C) serum analyzed by ELISA (N = 4–6/group) and immunoblot (N = 4/group). (D) Representative immunohistochemical images (scale bar: 45 μm) (black square scale bar: 25 μm) of CHOP-stained nuclei (black arrowheads), with quantification graph depicting nuclear H-score from 10 images from PAK2 mice hearts. (E) Immunofluorescence staining (scale bar: 50 μm) showing cellular localization of CHOP (red) and HMGB1 (green) following HFHG in H9C2. Lysosomes and nuclei are stained with LysoTracker (red) and DAPI (blue), respectively, and yellow puncta denote HMGB1 and lysosome colocalization. Relative CHOP nuclear intensity (AU) from 100 nuclei/data point (N = 4). (F) Schematic figure showing CHOP-binding sites in the promoter region (−8000 bp from transcription start site, TSS) in rat, human, and mouse *Hmgb1*. ChIP performed on H9C2s using anti-CHOP antibody (normalized to the input chromatin, N = 4). Primer pair 1 product sequence is highlighted gray; forward (F) and reverse (R) primer sequences flanking the corresponding region are annotated as Red = Primer one; Orange = Primer 2 and Green = Primer 3. (G) Strategy for macrophages stimulation using the conditional medium culturing H9C2s. (H–L) Immunoblot from H9C2s and conditional medium from (H and I) CHOP overexpression (N = 5) and (K and L) HFHG stimulation following PAK2 knockdown using siPAK2 (N = 3-5 experiments). Conditioned medium was analyzed for release of acetyl-HMGB1. Gβ and Coomassie stain are loading controls. (M and N) Representative flow contour plots and quantification of %CD206^+^ and %CD86^+^ macrophages with boxed mean M1/M2 ratio for BMM (chow-fed mice) treated with culture medium of H9C2 with (J) CHOP overexpression, (M) siScramble/PAK2 with HFHG (8 h) stimulation, and (N) Ad-Control/Ad-PAK2 with HFHG (12 h) stimulation. All M1/M2 ratios are calculated from mean %CD206^+^ and %CD86^+^ cells gated from parent (Ly6G^−^F4/80^+^) macrophages. Each data point represents one animal/experiment. All IP experiments (N = 1/group/cohort). All data are presented as mean ± SEM. *p* values (shown in each panel) versus corresponding controls determined by two-tailed Student's *t* test in (C, D, F, H, and I) and by ANOVA with Tukey's/Sidak's post hoc tests in (B, C, E, and J–N). Pearson R coefficient analysis in (A).

Of note, the negative correlation between cardiac *Hmgb1* and PAK2 (Figure 5A) warranted further investigation on how ER dysfunction induces myocardial HMGB1 under diabetic stress. Interestingly, cardiac *Pak2* ablation accelerated increase of HMGB1 at both transcript (Figure 3L) and protein level (Figure 5B) post HFHSD feeding. Likewise, *Pak2* depletion gave rise to a corresponding increase in intracellular and extracellular-acetylated HMGB1 (Figures 5B and 5C). We then asked how ER dysfunction induces HMGB1 gene upregulation in response to diabetic stress. CHOP plays a multifunctional role in the transcriptional regulation of genes (Nishitoh, 2012). It was increased in the HFHG-stimulated human heart slices and isolated rat cardiomyocytes (Figures S10A and S10B). Similarly, CHOP expression and nuclear localization were more pronounced in the *Pak2*^*cKO*^ myocardium in diabetes (Figures 3H and 5D). In addition, higher nuclear CHOP and concurrent shuttling of HMGB1 from the nucleus to the cytosol was detected in cells following 12-h HFHG stimulation (Figure 5E). Subsequently, we investigated whether CHOP transcriptionally regulates HMGB1. *In silico* screening of *Hmgb1* promoter region detected multiple potential binding sites of CHOP, which are highly conserved across mammalian species (Figure 5F). CHOP transcriptional regulation of HMGB1 was corroborated by chromatin immunoprecipitation (ChIP)-qPCR assay. CHOP overexpression led to a notable increase in the occupancy of CHOP to *Hmgb1* promoter regions containing its consensus binding sequence (−127 to −202 and −4366 from TSS) (Figure 5F). To validate cardiac stress-mediated inflammation, conditional medium from stimulated H9C2 was used to culture BMM (Figure 5G). Reinforcing ChIP findings, CHOP overexpression in H9C2 exhibited higher HMGB1 and its acetylated form (Figure 5H), leading to its subsequent secretion into the culture medium (Figure 5I). Next, stimulating mouse BMM with H9C2 culturing medium from *Chop*-overexpressing cells promoted M1 macrophage phenotype, confirming CHOP's role in HMGB1-mediated inflammation (Figure 5J). More supportively, mirroring the diabetic *Pak2*^*cKO*^ mice, *Pak2* abrogation in H9C2s increased CHOP accompanied by higher HMGB1 and its lysine-acetylated form upon HFHG (Figure 5K), with an elevated HMGB1 secretion (Figure 5L). Consequently, the conditioned medium from *Pak2*-knockdown H9C2s followed by stimulation of HFHG amplified M1/M2 ratio of BMM (Figure 5M). Consistently, M1 macrophage phenotype was repressed with conditioned medium from *Pak2*-overexpressing H9C2s followed by HFHG stimulation (Figure 5N). In summary, these data unmasked that cardiac HMGB1 expression and secretion is upregulated by means of exaggerated ER stress response-increased CHOP under diabetic conditions.

### PAK2-mediated functional ER is a requisite for vildagliptin's action on myocardial inflammation under diabetic conditions

**Figure 6:**
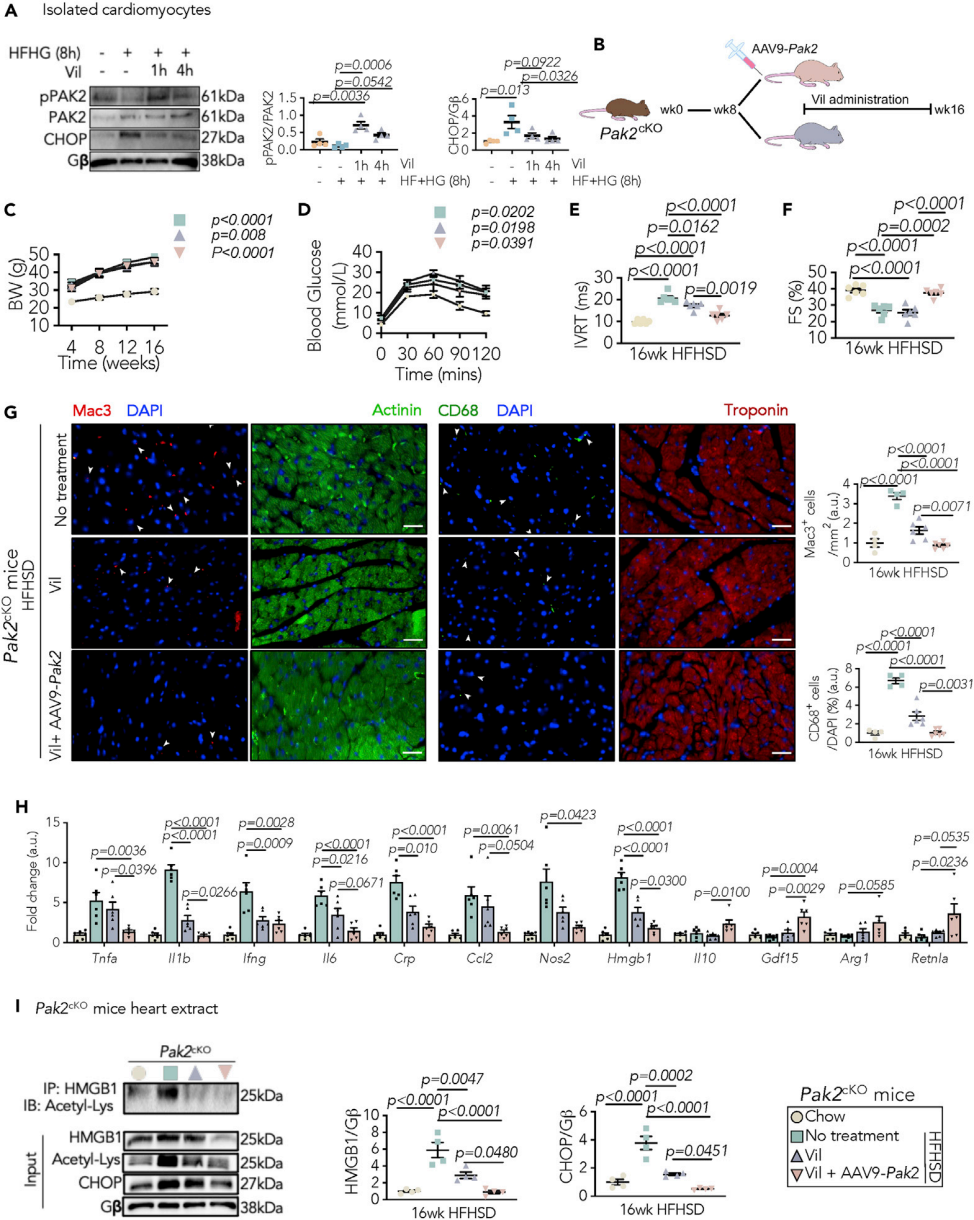
A retained ER function is a requisite for anti-inflammatory action of vildagliptin under diabetes (A) Representative immunoblot images and quantification of PAK2 activation and CHOP expression from isolated ARCM in the fact of vildagliptin (Vil) (20 μM) with diabetes-mimicking stress (N = 4). (B) Strategy for Vildagliptin administration (5 mg/kg/day in drinking water) with or without application of AAV9-*Pak2* in *Pak2*^*cKO*^ mice fed with HFHSD. (C and D) (C) BW (D) intraperitoneal glucose tolerance test. (E and F) (E) IVRT from pulse-wave Doppler analysis and (F) calculated FS from M-mode echocardiography (N = 6 in C–F). (G) Representative immunofluorescence staining (scale bar: 25 μm) and quantification of *left:* Mac3- (red) and *right:* CD68- (green)-positive non-cardiomyocytes in heart sections. Cardiomyocytes are counterstained with α-actinin (green) and troponin (red). Nuclei are stained with DAPI (blue) (N = 4/group). (H) Relative mRNA expression (normalized to chow, AU) of pro- and anti-inflammatory markers in *Pak2*^*cKO*^ hearts (N = 5–6/group). (I) Immunoprecipitation of acetylated HMGB1 from protein lysate. Immunoblot images and quantification of CHOP and HMGB1 (N = 4/group). Gβ is loading control. *Inset: key for data points*; each data point represents one animal/experiment. All data are presented as mean ± SEM. *p* values (shown in each panel) versus corresponding controls, determined by ANOVA with Tukey's post hoc tests in (A and C–I).

Given that diet-induced diabetic *Pak2*^*cKO*^ myocardium exhibited impaired cardiac ER function with a severe inflammatory response, we hypothesized that manipulating ER function has therapeutic potential for preventing myocardial inflammation in diabetes. We initially examined whether the current anti-diabetic medications regulate ER pathway, including empagliflozin (sodium-glucose co-transporter 2 inhibitor), semaglutide (glucagon-like peptide-1 receptor agonists), and vildagliptin (dipeptidyl peptidase-4 inhibitor). Among them, vildagliptin retained PAK2 activation and lessened HFHG-induced CHOP during short-term stress, while the others failed to function on managing ER stress via PAK2 (Figure S11A). The action of vildagliptin on ER normality was further confirmed in isolated cardiomyocytes and myocardium (Figures 6A, S11B, and S11C). It has also been demonstrated that vildagliptin exhibits an anti-inflammatory potential in diabetes (Kagal et al., 2017). Moreover, vildagliptin has been reported to exert cellular protective roles by alleviating aggravated ER stress in hepatocytes and β-cells (Ma et al., 2018a; Shimizu et al., 2012). Therefore, we acquired functional evidence on whether vildagliptin restricts myocardial inflammation by mitigating ER stress in diabetes. The cardiac ER dysfunction mice (*Pak2*^*cKO*^ mice) were fed with HFHSD for 8 weeks, followed by vildagliptin treatment (Figure 6B). Here, we observed that moderate vildagliptin administration did not affect the BW or glucose tolerance (Figures 6C and 6D). Also, it had no significant impacts on cardiac contractility in *Pak2*^*cKO*^ hearts (Figures 6E and 6F; Table S4). It is noteworthy that even though vildagliptin leads to an increase in left ventricular volumes in pre-clinical and clinical studies, it has no major effects on cardiac contractility (McMurray et al., 2018). Considering that cardiac PAK2 was abolished during diabetes development, we postulated that restored PAK2 serves as a molecular basis for supporting vildagliptin-induced ER regulation. Therefore, a combination of vildagliptin with PAK2 restoration using a gene delivery system was subjected to *Pak2*^*cKO*^ mice fed with HFHSD. Strikingly, following cardiac PAK2 restitution, vildagliptin treatment attenuated cardiac dysfunction post HFHSD, as demonstrated by reduced IVRT and reserved contractility compared to solely vildagliptin-treated mice (Figures 6E and 6F; Table S4). Pathological remodeling was also diminished by treatment of vildagliptin in the presence of PAK2, whereas standalone vildagliptin treatment gave rise to only a minor improvement (Figure S12).

More importantly, the elevated macrophages in the myocardium, identified by CD68 and Mac3, were significantly reduced by vildagliptin only in the attendance of cardiac PAK2 (Figure 6G). Further analyses of myocardial inflammatory profiles revealed that the pro-inflammatory cytokines, including *Tnfa*, *Il1b*, *Ifng*, *Il6*, and *Crp*, were significantly impeded by vildagliptin combined with cardiac PAK2 restoration (Figure 6H). Also, the dominant M1 macrophage subtype was lowered, which was evidenced by decreased *Nos2* and *Hmgb1* (Figure 6H). On the contrary, the markers of M2 macrophage subtype, such as *Il10*, *Gdf15*, and *Retnla*, were further enhanced particularly by the combined treatment (Figure 6H). Because macrophage polarization and inflammatory response are likely attributable to cardiac ER stress-induced HMGB1, we finally examined whether PAK2 existence is required for vildagliptin's anti-inflammatory function. Vildagliptin was able to considerably suppress the levels of cardiac CHOP and HMGB1 in the presence of PAK2 (Figure 6I). Collectively, these results unveil that regulated ER stress in cardiomyocytes is necessary for the net beneficial effects of vildagliptin on myocardial inflammation.

### Blocking extracellular HMGB1 decelerates deleterious effects of myocardial inflammation on DCM development

**Figure 7:**
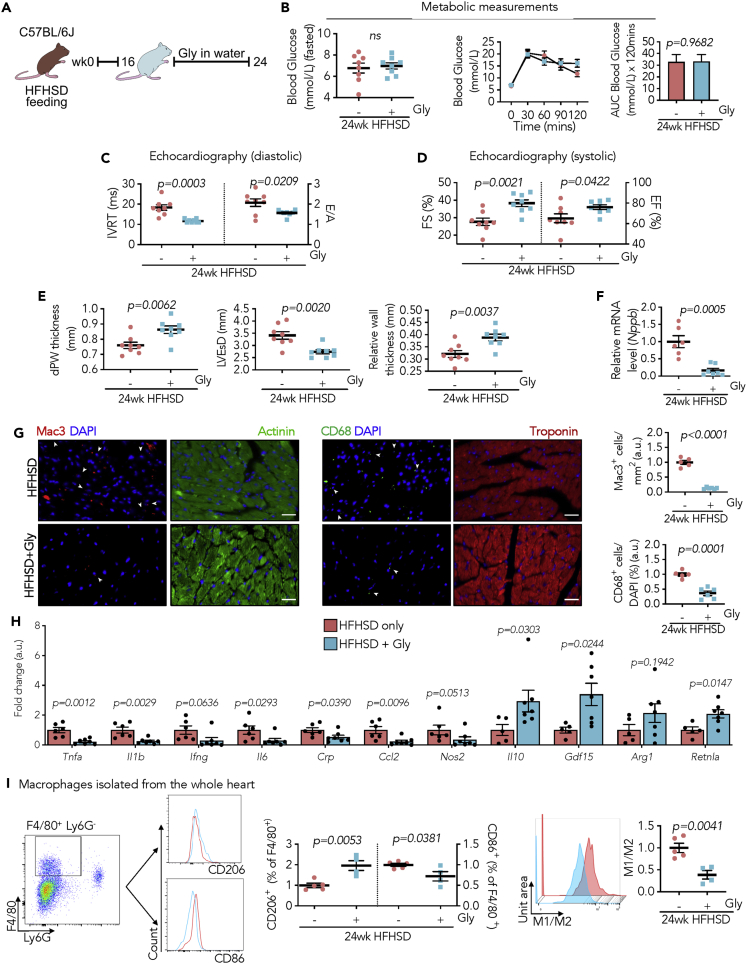
Glycyrrhizin prevents cardiac inflammation and decelerates DCM development by antagonizing extracellular HMGB1 (A) Experimental overview of treating with (+Gly) and without (-Gly) glycyrrhizin (150 mg/kg/day in drinking water). (B) Fasted blood glucose and intraperitoneal glucose tolerance test, and bar graph representing AUC (N = 8/group). (C) IVRT *(left)* and E/A ratio (*right*) (N = 8/group). (D and E) (D) FS *(left)* and EF (*right*), (E) dPW, LVEsD, and relative wall thickness (N = 8/group). (F) Relative mRNA expression of *Nppb* (-Gly normalized to 1, AU) (N = 5–7/group). (G) Representative immunofluorescence staining (scale bar: 25 μm) and quantification of Mac3- (red) and CD68- (green)-positive non-cardiomyocytes in heart sections. Cardiomyocytes are counterstained with α-actinin (green) and troponin (red). Nuclei are stained with DAPI (blue) (N = 5–6/group). (H) Relative mRNA expression (-Gly normalized to 1, AU) of pro- and anti-inflammatory markers (N = 5–7/group). (I) Representative flow cytometry gating (Figure S14A) for myocardial macrophages (CD45^+^Ly6G^−^F4/80^+^) and histogram displaying CD206 and CD86 macrophage subtypes. %CD206^+^*(left)* and %CD86^+^ (*right*) macrophages from CD45^+^Ly6G^−^F4/80^+^ cells. Representative histogram displaying derived parameter (M1/M2) vs. unit area (AU), calculated using derive parameter function on FlowJo from corresponding fluorescent channels of M1 (CD86) and M2 (CD206) (N = 4–5/group). Each data point represents one animal. All data are presented as mean ± SEM. *p* values (shown in each panel) determined by two-tailed Student's *t* test.

Extracellular HMGB1 has been considered as a potential therapeutic target in inflammatory diseases (Andersson et al., 2018). Finally, we ascertained functional evidence whether targeting HMGB1 has beneficial effects on diabetes-related cardiac dysfunction through anti-inflammatory activity. Glycyrrhizin is a natural compound that binds HMGB1 and inhibits its cytokine activities (Deutch et al., 2019). To seek its effects on DCM, C57BL/6J mice were fed with HFHSD for 16 weeks, the time point at which cardiac HMGB1 secretion rose, followed by glycyrrhizin administration in drinking water for further 8 weeks (Figure 7A). Although blockage of extracellular HMGB1 did not affect the systemic metabolic profiles, evidenced by hyperglycemia and glucose intolerance (Figure 7B), glycyrrhizin rescued cardiac function (Figures 7C and 7D; Table S5) and ameliorated cardiac pathological remodeling with reduced *Nppb* levels (Figures 7E, 7F, and S13). We further appraised whether prevention of DCM development is, at least partly, through abrogating inflammatory signatures in the myocardium. Macrophage prevalence in the myocardium was reduced by glycyrrhizin as confirmed by Mac3 and CD68 immunofluorescence staining (Figure 7G). Most importantly, examination of the inflammatory cytokines in the myocardium confirmed that pro-inflammation was diminished, shown with a decrease in *Tnfa*, *Il1b*, *Il6*, *Crp*, *and Ccl2* (Figure 7H). In addition, the genes featuring the M1 macrophage phenotype, such as *Nos2,* were decreased, while M2 macrophage markers, including *Il10*, *Gdf15*, and *Retnla*, were maintained in the myocardium from mice with glycyrrhizin administration (Figure 7H). In a further demonstration, macrophages were isolated from the heart and sorted as CD45^+^Ly6G^−^F4/80^+^ cells (Figure S14A). The percentage of macrophages that originated from either the M1 or M2 sub-population were labeled CD86^+^ or CD206^+^, respectively. M1 macrophages, as the major type of macrophages observed in the diabetic heart, were constrained after antagonizing copious HMGB1 using glycyrrhizin (Figure 7I). Finally, the whole heart extracts were examined with a Mouse cytokine array assay of 96 factors, which revealed a decrease in CD40, CTACK, CXCL1, MIP1γ, and sTNFRI, also supporting that glycyrrhizin halts myocardial inflammation in diabetes (Figure S14B). Taken together, these outcomes demonstrate that neutralizing HMGB1 hampers myocardial inflammation, which consequently slows down the progression of DCM and restores cardiac capacity to withstand chronic metabolic stress.

## Discussion

This study highlights the therapeutic notion of regulating cardiac ER stress response-associated inflammation in DCM. We dissect that PAK2 is a regulator of ER stress response in diabetes with an increase in CHOP because of the dysregulated ER response. Acting as a transcription factor, CHOP directly upregulates HMGB1, subsequently resulting in its active secretion from cardiomyocytes. Myocardial inflammation is further aggravated from cardiomyocyte-sourced HMGB1 activation of pro-inflammatory macrophages. In addition, as functional evidence, we showed that un-mitigated cardiac ER response due to PAK2 loss under diabetes may account as a barrier for leveraging the anti-inflammatory potential of vildagliptin. Also, sequestering extracellular HMGB1 restricts the excessive inflammatory reaction and decelerates DCM development.

### Cardiac Pak2 loss induces maladaptive ER stress in response to metabolic stress

The overactivated PERK and ATF6 branches of the UPR signaling cascade have been implicated in DCM development in rodent hearts (Lakshmanan et al., 2013). On the contrary, XBP1s is reported to be protective in type 1 diabetic mice hearts (Wang et al., 2021) and metabolic stress-induced HF with preserved EF model (HFpEF). The latter model driven by high-fat diet in combination with endothelial dysfunction-based hypertensive stress (Schiattarella et al., 2019) displayed a decline in myocardial XBP1s associated with reduced diastolic cardiac performance. This is consistent with our observations that ER dysfunction is evident with decreased XBP1s and increased XBP1u in the failing heart from diabetic patients and HFHSD-fed C57BL/6J mice. Moreover, cardiomyocyte-restricted overexpression of XBP1s rescued the HFpEF phenotype (Schiattarella et al., 2021), highlighting the protective role of adaptive UPR. As illustrated here, dysregulated ER response following PAK2 ablation is associated with early diastolic dysfunction and late decreased contractility following metabolic stress. We previously discovered that cardiac PAK2-mediated XBP1s restores ER homeostasis in response to pressure overload stress (Binder et al., 2019). The increase in ATF4 and ATF6 levels along with a reduction in PAK2 in diabetic myocardium from mice and humans, suggests that inhibition of XBP1s results in an exacerbated ATF4- and ATF6-mediated CHOP upregulation. Given the plethora of DCM instigators and temporal nature of the ER stress response, there is potential therapeutic significance in re-establishing ER homeostasis.

### ER stress directly triggers inflammatory response

Under pathological conditions, prolonged ER stress, as in diabetes, induces CHOP-dependent maladaptive effects (Doroudgar and Glembotski, 2013; Minamino et al., 2010). Accumulating data demonstrate a clear connection between ER stress and inflammation in a vicious cycle contributing to disease pathogenesis (Zhang and Kaufman, 2008). For instance, IRE1 and PERK branches activate the nuclear factor kappa B upregulation of inflammatory genes (Garg et al., 2012). In particular, CHOP facilitates the expression of pro-inflammatory cytokines, such as IL1β, IL8, and IL23, in either immune cells or non-immune cells (Endo et al., 2006; Goodall et al., 2010; Park et al., 2010). Consequently, the ER stress-induced inflammatory reaction further propagates underlying pathological outcomes. The current dogma regarding CHOP in cardiomyocytes is aligned with its transcriptional action on apoptotic genes facilitating cardiac apoptosis, pathological remodeling, and heart failure (Fu et al., 2010; Okada et al., 2004). Of importance, myocardial inflammation following ischemia-reperfusion injury is reduced in CHOP-KO mice (Miyazaki et al., 2011). Additionally, in response to LPS, IL6 failed to increase in CHOP-deficient cardiomyocytes (Miyazaki et al., 2011). Our study affirms that the detrimental ER stress response gave rise to increased CHOP followed by aggravated inflammatory responses, which highlights cardiac ER stress as a contributing factor to inflammation in the stressed myocardium.

### Phenotype of macrophages affects cardiac function

Macrophage phenotypes may contribute to myocardium homeostasis or disease. Myocardial macrophages have two origins: cardiac-resident macrophages and infiltrating circulatory monocytes (Ma et al., 2018b). Given the increase in macrophages in the diabetic heart, the fact that the BMMs were activated in the conditional medium from cultured cardiomyocytes following HFHG, potentially implies the activation of blood-borne macrophages *in vivo*. Moreover, it is reported that hyperglycemia alters the BMM phenotype and imposes a high CVD risk (Edgar et al., 2021). Interestingly, the BMMs from diabetic mice retained these pro-inflammatory and -atherogenic characteristics, even when cultured under physiological glucose levels. Similar pro-inflammatory phenotype was observed in the BMMs from HFHSD-fed mice under basal state. Therefore, it is hypothetically suggested that the increased susceptibility of BM-sourced macrophages to M1 polarization participates in the exacerbated myocardial inflammation observed in the experimental DCM models. A concept is emerging whereby manipulating macrophage polarization is key to developing macrophage-mediated therapeutics. Clinical studies have demonstrated that circulating monocytes isolated from diabetic patients are vulnerable to activation into pro-inflammatory M1 subtype (Nikiforov et al., 2017), which is in line with our observation that BMMs from diabetic mice were more susceptible to polarization to M1 macrophages. Moreover, conditional medium from CHOP-overexpressed cells drove the induction to M1 phenotype, akin to the observation that CHOP facilitates CCL11- and CCL24-mediated M1 polarization in adipose tissue (Suzuki et al., 2017). M1 macrophages are known to produce cytokines, such as TNFα, IL1β, IL6, CCL2 (MCP1), and CCL5, which were upregulated in the diabetic myocardium in our study. It is speculated that some cytokines (e.g. IL1β and IL6) were originated from cardiomyocytes at an early diabetic stage, followed by an even greater increase in cytokine levels due to both stressed cardiomyocytes and activated M1 macrophages. Noticeably, signatures of M2 macrophages (*Arg1*, *Retnla*, and *Ym1*) and their featured cytokines (IL10 and IL15) were predominant at an early stage, indicating that the M2 macrophages-mediated anti-inflammatory response was declined upon long-term diabetic stress. On the other hand, the pro-fibrotic role of M2 macrophages was presented by increased *Tgfb* along with decreased *Mmp9* (Braga et al., 2015), as a consequence of increased fibrosis in the diabetic heart. Therefore, these results support the previous hypotheses that an imbalance in macrophage polarization is associated with DCM development (Bajpai and Tilley, 2018).

### Regulation of cardiac intracellular HMGB1

HMGB1 is a non-histone chromosome-binding protein which participates in gene regulation; however, it acts as a DAMP promoting inflammation once released from inflammatory and stressed cells (Magna and Pisetsky, 2014). Although cardiac nuclear HMGB1 has a protective role in preventing pathological remodeling (Funayama et al., 2013), exogenous HMGB1 enhances pressure overload-induced cardiac hypertrophy (Zhang et al., 2016, 2019). Importantly, cardiomyocyte-specific HMGB1 deletion causes metabolic disorder in the myocardium (Yu et al., 2020). Funayama et al. observed that nuclear HMGB1 expression is decreased in the failing heart, whereas its cytoplasmic level is enhanced in response to neurohumoral stress (Funayama et al., 2013). Consistent with this, we detected HMGB1 translocation from nuclei to cytosol upon the change from compensated to decompensated state of diabetic stress in cardiomyocytes. It is worth recognizing that the molecular mechanisms that control HMGB1 expression in cardiomyocytes under pathological stress would be first described by this study. Evidently, forced expression of CHOP upregulated HMGB1 expression in cardiomyocytes. More convincingly, ChIP assay determined the direct binding of CHOP to the proximal promoter regions of HMGB1. Likewise, CHOP-regulated HMGB1 was also demonstrated in renal tubular cells (Zhang et al., 2015). Hence, HMGB1 is a novel target transcriptionally regulated by CHOP, which could bridge the knowledge gap regarding ER dysfunction-mediated cardiac inflammation; however, whether CHOP is a feasible target for preventing inflammation in the myocardium needs to be evaluated.

### HMGB1 is secreted from stressed myocardium

With regards to HMGB1 secretion, it occurs via ER-independent secretory pathways and depends on its post-translational modifications such as acetylation and its redox state, specifically disulfide bonds (Andersson et al., 2021; Kwak et al., 2020). In immune cells, its cytoplasmic translocation and subsequent release require acetylation in the nuclear localization sequence (Magna and Pisetsky, 2014; Ramadan et al., 2017) via Janus kinases/signal transducer and activator of transcription-1 or poly(ADP-ribose) polymerase-1 pathway (Lu et al., 2014; Yang et al., 2014). Of note, we found that myocardial HMGB1 was upregulated prior to macrophage appearance, indicating its possible source from non-immune cells. Its increase was also detected earlier than marked cardiomyocyte death. HMGB1 levels in the serum and the myocardium are higher in patients with T2D than in non-diabetic subjects (Volz et al., 2010; Zhao et al., 2013). In agreement with this fact, we demonstrate that the secretory form of HMGB1 was rising in the diabetic heart from both humans and mice. Such an increase was also observed due to CHOP overexpression, suggesting that HMGB1 can be secreted actively from stressed cardiomyocytes. However, the molecular basis of how CHOP mediates cardiac HMGB1 acetylation and translocation will be further investigated.

### Extracellular HMGB1 can be targeted for dampening of myocardial inflammation

Because secreted HMGB1 recruits and activates pro-inflammatory macrophages through TLR4 or the receptor for advanced glycation end products (RAGE) (Volz et al., 2010), we found an increased ratio of M1/M2 induced by rHMGB1 under diabetic stress. Moreover, induction of phenotypic switching to M1 macrophage polarization via HMGB1-TLR2/TLR4 signaling cascade has been previously associated with age-related cardiac dysfunction (Karuppagounder et al., 2016) and autoimmune myocarditis development (Su et al., 2016). Therefore, repression of extracellular HMGB1 appears to be an attractive treatment approach for decelerating myocardial inflammation without any negative effects on cardiomyocytes (Palomer et al., 2018). Previous studies illustrated that glycyrrhizin displays cardioprotective actions (Bangert et al., 2016; Cai et al., 2017). More importantly, glycyrrhizin also ameliorates insulin resistance, hyperglycemia, and hyperlipidemia in obesity and diabetes (Alizadeh et al., 2018; Sen et al., 2011). Here, we show that diabetes-associated myocardial inflammation, as assessed by cytokine levels and markers of macrophage subtypes, was attenuated by sustained administration of glycyrrhizin. These findings also explicate the previous finding that glycyrrhizin alleviates cardiac remodeling in the diabetic rat (Thakur et al., 2021). However, time will tell whether its beneficial effects can persist with meticulous investigation.

### Synergistic effects of antidiabetic drug and cardiac Pak2 maintenance in DCM

Finally, vildagliptin, a dipeptidyl peptidase-4 inhibitor, maintains incretin action in producing more insulin. In clinical use, a combination of vildagliptin and metformin has been approved as the first-line treatment for T2D (Das et al., 2020). Although pre-clinical research demonstrates that vildagliptin attenuates cardiac dysfunction (Li et al., 2021; Rankovic et al., 2021), clinical studies did not show compelling evidence of any beneficial cardiac effects (Das et al., 2020). Moreover, vildagliptin is capable of inhibiting free-fatty acid-induced HMGB1 release from endothelial cells (Qi et al., 2019). Here, we show that vildagliptin only participated in PAK2 phosphorylation with no effect on its expression. In consequence, vildagliptin minimized the ER dysfunction-induced CHOP expression solely with preserved PAK2 levels in the heart. Pre-eminently, we used a moderate dose of vildagliptin to obtain functional evidence for its impacts on cardiac inflammation. We observed that the cardiac PAK2 deficiency-associated maladaptive ER response restricted vildagliptin's inhibitory effect on CHOP-upregulated HMGB1 expression and thereby myocardial inflammation. In contrast, PAK2 maintenance was required to restore an intact ER response following vildagliptin treatment, in turn suppressing CHOP. As a consequence, cardiac HMGB1-amplified inflammation was diminished under diabetic stress. Prompted by the decline in cardiac PAK2 in long-term diabetes, our data provide a hypothetical explanation for clinical outcomes where vildagliptin has a limited effect on cardiac function due to its futile effect on PAK2 activation. Furthermore, our study suggests that ER function regulators as an adjunct to clinical antidiabetic interventions may have synergistic effects on both myocardial inflammation and DCM progression.

## Conclusion

In summary, we identify a new cardiomyocyte-macrophage communication in a cardiac ER dysfunction-dependent manner, altering the macrophage response in the myocardium. Specifically, PAK2 loss disrupted ER homeostasis and led to upregulation of cardiac-sourced HMGB1 via CHOP, profoundly affecting myocardial inflammation under metabolic stress. According to the results reported here, maintaining cardiac ER function could be a potential option, particularly in conjunction with antidiabetic medications, for limiting cardiomyocyte-related paracrine inflammation. Hence, future strategies aimed at the maintenance of cardiac ER function might be a feasible therapeutic approach to mitigate inflammation, thus alleviating cardiac dysfunction in patients with diabetes.

### Limitations of study

Our findings indicate that HMGB1 derived from stressed-cardiomyocytes induces pro-inflammatory effects on polarization of macrophages. However, at present, we lack direct evidence of the source of HMGB1 in serum during different time durations of metabolic stress, although *in vitro* findings established that cardiomyocytes are one source. Thus, the possible effects of cardiomyocyte-derived HMGB1 on non-myocytes during DCM might also be explored. Moreover, it can be useful to distinguish whether the activated macrophages in the diabetic myocardium are systemic or resident. Further investigations are required to understand the mechanisms of the interactions between cardiomyocytes and macrophages. In addition, although data from cells have clearly shown that CHOP overexpression upregulates HMGB1 expression, hyperacetylation, and subsequent secretion, genetically modified models with CHOP overexpression or knockdown would be better to affirm our findings. Additionally, we should also investigate the in-depth molecular mechanisms of HMGB1 intracellular translocation, specific acetylation sites, and secretion pathways from cardiomyocytes. Also, our study quantified hyperacetylated isoform of HMGB1 in human and mouse serum samples; it is worthwhile investigating the other isoforms of HMGB1, such as redox state, for a better understanding on the role of extracellular HMGB1. Metabolic stress induces HF with reduced and/or preserved EF. In this study, cardiomyocyte-specific PAK2 knockout model exhibited HF with reduced EF under such a stress. As a result, other ER dysfunction models and large animal models with metabolic disorders, including HF with preserved EF, could be utilized to prove the molecular basis for the paracrine effects of stressed cardiomyocytes on myocardial inflammation, thereby enhancing the therapeutic potential of the current findings.

## STAR★Methods

### Key resources table

**Table:** 

REAGENT or RESOURCE	SOURCE	IDENTIFIER
**Antibodies**		

Acetyl-HMGB1: 1:1000 in 5% milk	Antibodies.com	Cat# A51690; RRID:AB_2895269
ATF4: 1:1000 in 5% milk	Proteintech	Cat# 10835-1-AP, RRID:AB_2058600
ATF6: 1:1000 in 5% milk	Abcam	Cat# ab37149, RRID:AB_725571
Caspase 12: 1:1000 in 5% milk	Abcam	Cat# ab62484, RRID:AB_955729
Cdc42: 1:1000 in 5% milk	BD Biosciences	Cat# 610929, RRID:AB_398244
CHOP: 1:1000 in 5% milk	Cell Signaling Technology	Cat# 2895, RRID:AB_2089254
eIF2α: 1:1000 in 5% milk	Cell Signaling Technology	Cat# 9722, RRID:AB_2230924
GRP78: 1:1000 in 5% milk	Abcam	Cat# ab21685, RRID:AB_2119834
GRP94: 1:1000 in 5% milk	Cell Signaling Technology	Cat# 2104, RRID:AB_823506
Gβ: 1:500 in 5% milk	Santa Cruz Biotechnology	Cat# sc-166123, RRID:AB_2109632
HMGB1: 1:1000 in 5% milk	Abcam	Cat# ab18256, RRID:AB_444360
IRE1: 1:1000 in 5% milk	Abcam	Cat# ab37073, RRID:AB_775780
PAK2: 1:1000 in 5% milk	Cell Signaling Technology	Cat# 2608, RRID:AB_2283388
phospho-eIF2α: 1:1000 in 5% milk	Cell Signaling Technology	Cat# 9721, RRID:AB_330951
PERK: 1:500 in 5% BSA	Cell Signaling Technology	Cat# 3192, RRID:AB_2095847
phospho-IRE1: 1:500 in 5% milk	Abcam	Cat# ab243665, RRID:AB_2895271
phospho-PAK2: 1:1000 in 5% milk	Cell Signaling Technology	Cat# 2607, RRID:AB_2158759
phospho-PERK: 1:1000 in 5% milk	Abcam	Cat# ab192591, RRID:AB_2728666
Rac1: 1:500 in 5% milk	Millipore	Cat# 07-1464, RRID:AB_1977451
XBP1 s/u: 1:500 in 5% milk	Abcam	Cat# ab37152, RRID:AB_778939
Acetylated lysine: 1:1000 in 5% milk	Abcam	Cat# ab22550, RRID:AB_447149
APC/Cyanine7 CD86: 1:100 for heart and bone marrow macrophages	BioLegend	Cat# 105029, RRID:AB_2074993
APC CD206 (MMR) :100 for heart and bone marrow macrophages	BioLegend	Cat# 141707, RRID:AB_10896057
AF700 Ly6G 1:200 for heart and bone marrow macrophages	BioLegend	Cat# 127621, RRID:AB_10640452
BV510 CD45 1:100 for heart macrophages	BioLegend	Cat# 103137, RRID:AB_2561392
PE F4/80 1:50 for heart and 1:500 for bone marrow macrophages	BioLegend	Cat# 123109, RRID:AB_893498
CD68: 1:100 in 0.1% Triton-X in PBS	Abcam	Cat# ab31630, RRID:AB_1141557
Mac3: 1:300 in 0.1% Triton-X in PBS	BioLegend	Cat# 108501, RRID:AB_31338
Troponin (T-C): 1:100 in 0.1% Triton-X in PBS	Santa Cruz Biotechnology	Cat# sc-8121, RRID:AB_2287642
α-actinin: 1:500 in 0.1% Triton-X in PBS	Sigma-Aldrich	Cat# A7811, RRID:AB_476766
Anti-mouse AlexaFluor488: 1:200 in 0.1% Triton-X in PBS	Jackson ImmunoResearch	Cat# 715-586-151, RRID:AB_2340858
Anti-goat AlexaFluor594: :200 in 0.1% Triton-X in PBS	Jackson ImmunoResearch	Cat# 705-585-147, RRID:AB_2340433
Anti-rat AlexaFluor594: 1:200 in 0.1% Triton-X in PBS	Jackson ImmunoResearch	Cat# 712-585-153, RRID:AB_2340689
Ly6G (1:100 in 0.1% TritonX in PBS)	BioLegened	Cat# 127602, RRID:AB_1089180
HRP-linked anti-mouse	Cell Signaling Technology	Cat# 7076, RRID:AB_330924
HRP-linked anti-rabbit	Cell Signaling Technology	Cat# 7074, RRID:AB_2099233
HRP-linked anti-rat	Abcam	Cat# ab102182, RRID:AB_10711694
CD16/32	BioLegend	Cat# 101319, RRID:AB_1574973

**Bacterial and virus strains**		

AAV9-*Pak2*	Laboratory of Xin Wang (Binder et al., 2019)	N/A
Adenovirus-PAK2	Laboratory of Xin Wang (Binder et al., 2019)	N/A

**Biological samples**		

Human myocardial protein extracts	Asterand (US lab, Hertfordshire, UK)	N/A

**Chemicals, peptides, and recombinant proteins**		

Glycyrrhizic acid/Glycyrrhizin	Sigma-Aldrich	Cat# G2137
Vildagliptin	Sigma-Aldrich	Cat# SML2302
Isoflurane	Isothesia, Henry Schein	N/A
Bouin's Solution	Sigma-Aldrich	Cat# HT10132
Harris' Hematoxylin	RA lamb Dry Chemical Stains	Cat# LAMB/230
Red Solution	Sigma-Aldrich	Cat# HT151
Aniline Blue	Sigma-Aldrich	Cat# B8563
Eukitt	Sigma-Aldrich	Cat# 03989
Eosin	Thermo Scientific	Cat# 6766007
VectaShield Antifade Mounting medium with DAPI	Vector laboratories	Cat# H-1000
Sudan Black	Sigma-Aldrich	Cat# 199664
DMEM containing 1 g/L glucose	Gibco	Cat# 11966-025
DMEM, high glucose	Gibco	Cat# 41965-039
Palmitic Acid	Sigma-Aldrich	Cat# P0500
Lipofectamine LTX	Invitrogen	Cat# 15300-100
Lipofectamine 2000 Reagent	Invitrogen	Cat# 11668-019
Emagliflozin	Cayman	Cat# 17375
Semaglutide	Cayman	Cat# 29969
DMEM+ Glutamax	Gibco	Cat# 21885
LysoTracker Red	Thermofisher	Cat# L7528
Bradford Assay	BioRad	Cat# 500-0006
Protein G Agarose beads	Cell Signaling Technology	Cat# 9007
Collagenase II	Worthington	Cat# LS004174
DNaseI	Worthington	Cat# LS002139
Human recombinant HMGB1	R&D	Cat# 1690-HMB
Lipopolysaccharide	Sigma-Aldrich	Cat# L2630
Murine recombinant IFNγ	BioLegend	Cat# 575302
Murine recombinant IL4	Antibodies.com	Cat# A21323
Flow cytometry staining buffer	eBioscience	Cat# 00-4222-26

**Critical commercial assays**		

Cholesterol Quantitation Kit	Sigma-Aldrich	Cat# MAK043
Rodent Insulin Chemiluminescence ELISA	Alpco	Cat# 80-INSMR-CH10
Mouse HMGB1 ELISA	Fine Test	Cat# EM0382
*in situ* Cell death detection kit	Roche	Cat# 11684795910
Cytokine Array – Mouse Cytokine Antibody Array (Membrane, 96 Targets)	Abcam	Cat# ab193659
LunaScript RT-PCR	New England Biolabs	Cat# NEB3010
SYBR Select PCR master mix	Applied Biosystems	Cat# 4472908
SimpleChip Plus Enzymatic ChIP Kit	Cell Signaling Technology	Cat# 9004
BCA protein assay kit	Thermo Pierce	Cat# 23225
ECL	Thermoscientific	Cat# 34087
ECL Prime	Amersham	Cat# RPN2232
ECL Select	Amersham	Cat# RPN2235

**Deposited data**		

Source Data	Mendeley	https://doi.org/10.17632/b5y5kvjdtt.1

**Experimental models: Cell lines**		

Primary bone-marrow derived macrophages from tibia and femur of C57BL/6J male mice	This paper	N/A
Primary adult rat ventricular cardiomyocytes from hearts of Sprague-Dawley rats	This paper	N/A
Human cultured heart tissue	Laboratory of Tamer Mohamed (Ou et al., 2019) and Novabiosis (USA transplantation network)	N/A
H9C2 cells	European Collection of Authenticated Cell cultures	Sigma-Aldrich, 88092904
L929 cells	ATCC	CCL-1

**Experimental models: Organisms/strains**		

Mouse: C57BL/6J	Envigo, UK	N/A
Rat: Sprague-Dawley Rats	Envigo,UK	N/A
Mouse: *Pak2*^*cKO*^ (C57BL/6J *Pak2*^*fl/fl*^*x aMHC-Cre)*	Laboratory of Jonathan Chernoff (Radu et al., 2015)	N/A

**Oligonucleotides**		

ChIP Primer 1 (bp from TSS -217 to −1) F: TCCTCGCAGACAGCCAATG R: GTCTCTATGGAGCTCAATGTACT	This paper	N/A
ChIP Primer 2 (bp from TSS -1548 to −1340) F: ACACCAATGATAGTCGCTAGACC R: AATCCAAGTCAAAACATTCAAGTCA	This paper	N/A
ChIP Primer 3 (bp from TSS -2585 to −2853) F: TCTCCCAATAAGCTTTGGCTGT R: GAGGGTAAGTTAATGGCCCACA	This paper	N/A
siScramble RNA: AGGUAGUGUAAUCGCCUUG	Sigma	N/A
Rat *Pak2* siRNA	Ambion	ID: s218097
Rat *Cdc42* siRNA	Ambion	ID: s134116
Rat *Rac1* siRNA	Ambion	ID: s171173
For qPCR primers see Table S6	Qiagen	N/A

**Recombinant DNA**		

CHOP plasmid	Source Bioscience	Cat# IRAVp968C1026D; NCBI accession BC013718, BF137987

**Software and algorithms**		

FlowJo	FlowJo LLC	https://www.flowjo.com/
GraphPad Prism 9	GraphPad Software	https://www.graphpad.com/scientific-software/prism/
ImageJ-Fiji	National Institutes of Health, USA	https://fiji.sc/
Imaris	Bitplane	https://imaris.oxinst.com/packages
LabChart 7	ADInstruments	https://www.adinstruments.com/support/software/archive
IHC profiler plugin	Varghese et al., 2014	N/A
Intensity Ratio Nuclei Cytoplasm Tool	GitHub	RRID: SCR_018573; https://github.com/MontpellierRessourcesImagerie/imagej_macros_and_scripts/wiki/Intensity-Ratio-Nuclei-Cytoplasm-Tool

**Other**		

High fat (45%) and high sucrose (20%) diet	Special Diet Services	Cat# 824018
Standard Chow diet	Special Diet Services	Cat# 801960

### Resource availability

#### Lead contact

Further information and requests for resources and reagents should be directed to and will be fulfilled by Wei Liu (wei.liu@manchester.ac.uk)

#### Materials availability

This study did not generate new unique reagents or mouse lines.

### Experimental model and subject details

#### Human heart samples

Human myocardial protein extracts from patients with diabetes and normal subjects were purchased from Asterand (US lab, Hertfordshire, UK). Ethical approval and consent were obtained by Asterand. The use of human tissue samples has been approved by the United Kingdom Human Tissue Authority.

#### Human serum samples

The human serum samples were collected, prepared, and provided by Dr Handrean Soran, which was approved by the Greater Manchester Research Ethics Committee. Venous blood samples were obtained between 0900 and 1100 following an overnight fasting. Serum was isolated by centrifugation at 4°C within 2 hours of collection and aliquots were stored frozen at −80°C until laboratory analyses performed at the end of study.

#### Study approval

All animal studies were performed in accordance with the United Kingdom Animals (Scientific Procedures) Act 1986 and were approved by the University of Manchester Ethics Committee.

#### Animals models

All mice and rats were housed in a pathogen-free facility at the University of Manchester. C57BL/6J mice and Sprague-Dawley rats were purchased from Envigo (UK). *Pak2*-floxed (*Pak2*^fl/fl^) mice were generated previously with two LoxP elements flanking exon2 of *Pak2* (Radu et al., 2015). Cardiomyocyte-specific *Pak2* knockout mice (*Pak2*^cKO^) were bred by mating *Pak2*^fl/fl^ mice with mice expressing Cre under a-myosin heavy chain (*aMHC*) promoter. *Pak2* deletion in cardiomyocytes was verified by PCR of genomic DNA using (5′-TGAAGCTGCATCAATCTATTCTG-3′) and (5′-TGAAGCTGCATCAATCTATTCTG-3′) primers. Mice were backcrossed into C57BL/6J background for 8 generations. *Pak2*^cKO^ and their littermates (*Pak2*^*f*l/fl^) were used in this study. When weaned, mice were randomly split into housing units containing three to six mice, blinded by the genotype. Each was considered an experimental unit and assigned feeding and treatment combinations. Each mouse was counted as a biological replicate. All *in vivo* studies were blinded for genotype and treatment during measurement and analysis. No exclusions were made. Mice were euthanized by cervical dislocation while sedated.

#### H9C2 cell culture

H9C2 cells were obtained from the European Collection of Authenticated Cell cultures (Sigma-Aldrich, 88092904). Prior to high fatty acid high glucose (HFHG) stimulation, cells were maintained in DMEM (Gibco, 11966-025) containing 1 g/L glucose and 1%FBS overnight. To mimic the effects of HFHSD *in vitro*, cells were exposed to HFHG medium (DMEM (Gibco, 41965-039) supplemented with 1%FBS, 500 μM palmitic acid (PA) (Sigma-Aldrich, P0500) in 0.5% BSA; 6 g/L glucose) for time points stated in the corresponding figures.

#### Adult cardiomyocytes (ARCMs) isolation and culture

6-wk-old male Sprague Dawley rats were sacrificed using a dose of pentobarbital (150 mg/kg) with 250 U of heparin. The adult cardiomyocytes were isolated as described previously (Ruiz-Velasco et al., 2020). The heart was removed and perfused retrogradely through the aorta. After washing with perfusion buffer (Ca^2+^ free Hank's buffered salt solution, 5.6 mmol/L glucose, 1 mmol/L MgSO4), collagenase was added (117 U/mL collagenase type 2, Worthington) followed by protease (0.1775 U/mL protease type XIV, Sigma). A 20-minute digestion was followed by cut up pieces of the left ventricle, which were further digested in a perfusion buffer containing collagenase and protease, along with 0.02 g/L trypsin and 0.02 g/L DNAse I, and 1 mmol/L CaCl2. Digested and filtered ARCMs were resuspended in ACCT mediums (DMEM+Glutamax medium (21885, Gibco) containing 1% BSA, 5 mmol/L L-carnitine, 2 mmol/L creatine, 5 mmol/L taurine, and 10 μmol/L blebbistatin). Finally, ARCMs were plated out and cultured in Geltrex (A1413302, Gibco) coated plates. Fresh ACCT medium was replaced every day before treatment. The isolated cardiomyocytes were subjected to HFHG stimulation following maintenance in DMEM containing (1 g/L glucose, 1% FBS) overnight. The cells were stimulated in HFHG medium (DMEM+Glutamax 21885, Gibco) containing 500 μM PA, 1%FBS, 2% BSA, 6 g/L glucose, 5 mmol/L L-carnitine, 2 mmol/L creatine and 5 mmol/L taurine) for 1, 4, 8 or 12 hs.

#### Human heart slices and culture

The cultured heart protein extracts were a kind gift from Dr. Tamer M.A. Mohamed (University of Louisville). Fresh human heart was provided from a consented and deidentified donor through the USA transplantation network ‘Novabiosis’. The protocol was approved by the IRB committee at the University of Louisville as a non-human subject research. The donor was aged 37 years old with no CVD history. The harvest and slicing of the heart are described previously (Ou et al., 2019). Briefly, freshly sliced heart was maintained in the culture medium overnight, followed by HFHG (500 μM PA in 0.5% BSA; 6 g/L glucose) stimulation for 16 hs.

#### Murine BM-derived macrophages culture

BM progenitors were isolated from male chow-fed 6-week-old C57BL/6J mice or mice fed HFHSD for 12 and 16 weeks. Following cervical dislocation, both hind legs were dissected and harvested. Tibia and femur were cleaned for excess tissue and rinsed in PBS. BM isolation was carried out with DMEM (11966-025, Gibco) supplemented with 1 mM sodium pyruvate using a 27G needle and 10 cc syringe. The flushed-out bone marrow was passed through a 19G needle to break down cell aggregates, filtered through a 70 μm strainer and centrifuged at 400 g for 10 mins. BM progenitors were resuspended in DMEM supplemented with 10% FBS, 1 mM sodium pyruvate and 20% L929 (CCL-1, ATCC, Middlesex, UK) conditioned media, containing macrophage-CSF. The resuspension was plated onto 100 mm sterile bacterial dishes at 14 million cells/dish and differentiated at 37°C and 5% (v/v) CO_2_ for 6 days, with additional feeding on day 3. On day 6, the BM-derived macrophages (BMM) were re-plated following trypsination for macrophages polarization.

### Method details

#### Feeding and treatment

6-week-old male C57BL/6J mice were randomly grouped into 8, 12, 16 and 24 weeks in a cross-sectional study using randomizer.com. In a longitudinal study, 6-8-week-old male *Pak2*^fl/fl^ and *Pak2*^cKO^ mice were randomly allocated and maintained on high fat high sucrose diet (HFHSD, 45% fat and 20% sucrose) (SDS, 824018) or standard chow (SDS, 801960) for 16 weeks.

Glycyrrhizic acid/Glycyrrhizin (a HMGB1 inhibitor, Sigma-Aldrich, G2137), and Vildagliptin (a DPP4 inhibitor, Sigma-Aldrich, SML2302) were administered to C57BL/6J mice and *Pak2*^cKO^ mice *ad libitum* in drinking water, at an estimated dose of 150 mg/kg/day and 3 mg/kg/day, respectively, along with HFHSD according to individual experimental design (see Results). The water pouch was changed every 3 days. For cardiac specific over-expression of PAK2, adeno-associated virus (AAV9-*Pak2*) was generated previously (Binder et al., 2019) using human *Pak2* cDNA (Source Bioscience, NCBI Accession # BC069613). The recombinant virus (0.5 × 10^11^ genomic particles) was administered via tail vein after 8 weeks of HFHSD feeding.

#### Echocardiography

For cardiac function evaluation, mice were anesthetized with 2% isoflurane (Isothesia, Henry Schein) mixed with 100% oxygen at 1.5 L/min rate. Transthoracic M-mode and pulse wave Doppler ultrasound images were obtained using Acuson Sequoia C256 system (Siemens) and Vevo 770 system (Visualsonics). For each mouse, left ventricle chamber and wall dimensions, diastolic function (IVRT, E/A) parameters, fractional shortening (FS%), ejection fraction (EF%), Left ventricular (LV) mass and relative wall thickness were measured or calculated.

#### Conscious ECG

Mice were placed on the platform and conscious 15 mins recordings were obtained and analyzed using LabChart 7 software. The average of 20 continuous beats was used to measure RR interval, P duration, QRS, corrected QT (QT_c_) and JT segment.

#### Metabolic measurements

Blood was collected from lateral tail vein, and serum was extracted for systemic measurements. Serum cholesterol (Sigma-Aldrich, MAK043), insulin (Alpco, 80-INSMR-CH10) and HMGB1 (Fine test, EM0382) levels were measured using kits according to the manufacturer's instructions. For glucose tolerance test (GTT), mice were fasted overnight, followed by *i.p.* injection of glucose injection (2 g/kg BW). Blood glucose levels were measured at 30 min intervals over the period of 2 hs using Accu-Chek Aviva (Roche) glucometer.

#### Transmission electron microscopy

Fresh heart samples were excised and fixed overnight in 0.1 M HEPES buffer (pH 7.2) containing 4% paraformaldehyde (PFA) and 2.5% glutaraldehyde, and then post-fixed in 0.1 M cacodylate buffer (pH7.2) with 1% osmium tetroxide and 1.5% potassium ferrocyanide for 1 h, followed by 1% tannic acid in 0.1 M cacodylate buffer (pH 7.2) for 1 h, finally treated in 1% uranyl acetate for 1 h. The samples were then dehydrated in ethanol and embedded in TAAB 812 resin and polymerized for 24 hs at 60°C. Sections were cut with Reichert Ultracut ultramicrotome and examined with FEI Tecnai 12 Biotwin microscope at 100 kV accelerating voltage. Images were taken with Gatan Orius SC1000 CCD camera.

#### Histology

The hearts were isolated and fixed in 4% v/w Paraformaldehyde (PFA), dehydrated, and cleared in xylene overnight, followed by paraffin embedding. The blocks were sectioned at 5 μm and 30 μm (for 3D imaging) thickness using Microtome (Leica). Heart sections were dewaxed and rehydrated prior to histological staining.

#### Masson's trichrome

Interstitial and perivascular fibrosis was analyzed by Masson’s Trichrome staining. The slides were immersed in Bouin's Solution (HT10132, Sigma-Aldrich) for 2 hs. This was followed by nuclear staining with Harris' Hematoxylin (LAMB/230, RA Lamb Dry Chemical Stains) for 5 mins and by differentiation in acid alcohol (1% hydrochloric acid, 70% ethanol) for 10 secs. Subsequently, sections were stained for cardiomyocytes and collagen using Red Solution (HT151, Sigma-Aldrich) and Aniline Blue (B8563, Sigma-Aldrich), respectively. Finally, sections were dehydrated in increasing concentrations of ethanol (75%, 95%, 100%) and cleared in xylene, and mounted with xylene-based medium, Eukitt (03989, Sigma-Aldrich).

#### Haematoxylin and eosin (H&E) staining

Cardiomyocyte cross-sectional area was calculated from H&E-stained myocardial paraffin sections. Following deparaffinization and rehydration, sections were incubated in Harris' Haematoxylin for 5 mins and differentiated with acid alcohol for 10 secs. The nuclei were counterstained with Eosin (6766007, Thermo Scientific) for 1 min. Samples were dehydrated, cleared, and mounted with Eukitt.

#### Immunohistochemistry

Paraffin-fixed heart sections were dewaxed and rehydrated in decreasing concentrations of ethanol (100%, 95%, 75%, 50%) for 5 mins each. The sections were immersed in 3% H_2_O_2_ in methanol for 10 mins to block endogenous peroxidase activity. Following heat antigen retrieval, the samples were blocked in 5% donkey/horse serum in 0.1% Triton-X in PBS, and then incubated with mouse anti-CHOP (1:100) and rat anti-Ly6G (BL 127602) (1:100) overnight at 4°C. The samples were washed and incubated with HRP linked anti-mouse (1:400) and anti-rat (1:400) (ab102182, Abcam) for 2 hs at room temperature. Following PBS washing, the signal was developed using diaminobenzidine (DAB) as peroxidase substrate and nuclei were counterstained Harris' Hematoxylin for 7 mins. Finally, the slides were dehydrated and mounted with Eukitt.

#### Immunofluorescence

Deparaffinized and rehydrated sections were heated in sodium citrate buffer (10 mM, pH6.0) at 95°C for 30 mins to allow antigen retrieval. Sections were blocked for 1 hr at room temperature with 10% donkey serum or BSA in 0.1% Triton-X in PBS. For immunofluorescence, mouse hearts were incubated with mouse anti-CD68 (Ab 31630, Abcam) (1:100), rat anti-Mac3 (1:300) (BL108501, BioLegend), goat anti-troponin (SC8121, Santa Cruz Biotechnology) (1:100) and mouse anti-α-actinin (A7811, Sigma-Aldrich) (1:500) primary antibodies overnight at 4°C. Following three washes in 0.1% Triton-X in PBS, tissues were incubated in donkey anti-mouse AlexaFlour488 (1:200), donkey anti-goat AlexaFlour594 (1:200), anti-mouse AlexaFlour488 (1:500) and anti-rat AlexaFlour594 (1:200) secondary antibodies for 1 h at room temperature. All staining steps were conducted in 5% donkey serum or BSA in 0.1% Triton-X in PBS. Finally, tissues were washed and mounted with VectaShield containing DAPI to stain the nuclei. For 3D reconstruction, samples were treated with 0.3% Sudan Black (199664, Sigma-Aldrich) in 70% ethanol for 1 h at room temperature for quenching autofluorescence prior to mounting.

#### Cytokine array

Antibody array targeting 96 mouse cytokines (ab193659, Abcam) was used to identify the inflammatory response in the mouse heart extracts following treatment with glycyrrhizic acid. Following the manufacturer’s instructions, the lysate was prepared by combining three mice ventricular tissues and subjected to the array at a final concentration of 4 mg/mL. Chemiluminescence was detected with a ChemiDocTM MP Imaging System (Bio-rad).

#### H9c2 gene knockdown or overexpression

Gene knockdown or overexpression in H9C2 was achieved with siRNA and transfection of cDNA using Lipofectamine LTX (Invitrogen, 15300-100) and Lipofectamine 2000 Reagent (Invitrogen, 11668-019), respectively. Cells were transfected with 100 nM of control siRNA (AGGUAGUGUAAUCGCCUUG, Sigma), rat *Cdc42* siRNA (Ambion ID: s134116), rat *Rac1* siRNA (Ambion ID: s171173) or rat *Pak2* siRNA (Ambion ID: s218097) following the manufacturer’s instructions. For CHOP over-expression, the cells were transfected with purified CHOP plasmid (Source Bioscience, IRAVp968C1026D; NCBI accession BC013718, BF137987) at 2 μg/mL for 48 hs PAK2 overexpression in cells was achieved by infecting the cells with adenovirus expressing PAK2 (Ad-PAK2) for 48 hs prior to the following stimulation (Binder et al., 2019). Vildagliptin, Empagliflozin (Cayman, 17375), and Semaglutide (Cayman, 29969) were added directly into the medium for pre-treatment.

#### Real-time quantitative PCR

Total RNA from tissue samples was extracted using Trizol. Samples were treated with DNAse (DNA-free Removal Kit, Invitrogen) to eliminate genomic DNA contamination. RNA was converted to cDNA using Lunascript (NEB3010, New England Biolabs). Specific primers (see Table S6) for quantitative real-time polymerase reaction (qPCR) were purchased from Qiagen and reactions were conducted using SYBR Select PCR Master Mix (4472908, Applied Biosystems) following the manufacturer’s instructions. qPCR reactions were run in the Step One Plus PCR System (Applied Biosystems), and fold change was computed by the comparative Ct (^ΔΔ^Ct) method. mRNA levels were normalized to 18S expression.

#### Immunocytofluorescence

To observe cellular localization of CHOP and HMGB1, following HFHG stimulation, H9C2 cells cultured on glass coverslips were fixed with 4% v/w PFA for 15 mins at room temperature. For staining lysosomes, the cells were treated with Lysotracker red (L7528, Thermofisher) for 2 hs at a concentration of 75 nM prior to fixation. The fixed cells were permeabilized with 0.1% Triton X-+ 0.1% sodium citrate for 10 mins. Coverslips were later blocked for 1 h in 10% v/v normal donkey serum (NDS) in 0.1% v/v Triton-X in PBS. Rabbit anti-HMGB1 and mouse anti-CHOP antibodies were diluted at 1:100 in 3% NDS in 0.1% Triton-X in PBS and applied to coverslips for incubation overnight at 4°C. A secondary donkey anti-rabbit AlexaFlour488 and donkey anti-mouse AlexaFlour594 antibodies at 1:1000 were used after washing with PBS. Finally, the coverslips were washed and mounted with VectaShield with DAPI.

#### Chromatin immunoprecipitation

Chromatin immunoprecipitation (ChIP) was performed using the SimpleChip Plus Enzymatic ChIP Kit (9004, Cell Signaling) as per manufacturer’s instructions. Briefly, control and CHOP-overexpressed H9C2 were fixed with 1% v/v formaldehyde and harvested. Nuclear membrane was lysed by sonication. Fragmented chromatin was immunoprecipitated by mouse anti-CHOP antibody. qPCR was subsequently performed using the following primer sets (See Key resources table) (Sigma-Aldrich) designed from rat *Hmgb1* (NC_051347) sequence. Data was normalized to input chromatin.

#### Protein purification from culture medium

The medium from treated H9C2 was collected and centrifuged at 200xg for 10 mins to remove cell debris. The medium protein was extracted by mixing 500 μL of medium with 500 μL of methanol and 125 μL of chloroform. Following centrifugation for 10 mins at 14,000 g, the aqueous layer was discarded, and the protein layer was dissolved in 500 μL methanol. The samples were then centrifuged at 14,000 g for 10 mins, and the protein was resuspended in ddH_2_O. Protein was quantified using Thermo Pierce BCA protein assay kit (23225). Coomassie gel stain was used as a loading control.

#### Immunoblotting from serum

The serum samples were diluted 1:200 (for mouse serum) and 1:100 (for human serum) in ddH_2_O. Coomassie gel stain was used as a loading control.

#### Protein lysates extraction and immunoblotting

Total protein lysates from tissue or cells were prepared with Triton lysis buffer (137 mmol/L NaCl, 20 mmol/L Tris, 0.1% w/v SDS, 2 mmol/L EDTA, 10% v/v glycerol, 1% Triton-X, 25 mmol/L glycerophosphate, 1 mmol/L Na_3_VO_4_, 1 mmol/L, 10 mM NAM, 1 μM TSA, 1xprotein cocktail inhibitor, pH7.4). Lysates were cleared by centrifuging for 20 mins at 14,000 g. Protein concentration was quantified by Bradford assay (500-0006, Bio-Rad). Immunoblot analysis was performed with 30 μg of protein lysate using the primary antibodies described in Key resources table. Secondary anti-mouse (7076, Cell Signaling) and anti-rabbit (7074, Cell Signaling) HRP conjugates were used along with the Thermoscientifc ECL (34087), Amersham ECL Prime and Select detection reagents (RPN2232 and RPN2235, Amersham) to detect chemiluminescence using ChemiDoc MP System (BioRad).

#### Immunoprecipitation

To investigate protein association, immunoprecipitations were performed with Protein G agarose beads (9007, Cell Signaling) following manufacturer’s instructions. Briefly, tissue was lysed with RIPA buffer (150 mmol/L NaCl, 50 mmol/L tris, 0.1% w/v SDS, 0.25% w/v sodium deoxycholate, 2 mmol/L EDTA, 5% v/v glycerol, 1% v/v Triton X-, 25 mmol/L glycerophosphate, 1 mmol/L Na_3_VO_4_, 1 mmol/L phenylmethanesulfonylfluoride, 1.54 μmol/L aprotinin, 21.6 μmol/L leupeptin, 10 mM nicotinamide, 5 μM TSA, pH7.4). Afterwards, 500 μg of the protein extract was cleared using G agarose beads for 2 hs. The beads were conjugated with rabbit anti-mouse HMGB1 antibody (3 μg/mg lysate) for 4 hs. The protein was immunoprecipitated using antibody-bead complex overnight at 4°C. Immune complexes were eluted in 2xLaemmli sample buffer (65.8 mM Tris-HCl, 2.1% SDS, 26.3% glycerol, 0.01% bromophenol blue, pH7.2). Precipitated and input proteins were subjected to SDS-PAGE and immunoblotted using mouse anti-mouse acetylated lysine (ab22550, Abcam) antibody.

#### Heart tissue single cell suspension

Hearts from HFHSD fed C57BL/6J mice with or without glycyrrhizin treatment were digested for flow cytometric analyses. The hearts were perfused through the abdominal aorta with HBSS. Following dissection, the hearts were harvested and chopped into small pieces. These were digested in 600 U/mL Collagenase II (215 U/mL, collagenase type 2, Worthington) and 60 U/mL DNase I (LS002139, Worthington) in DMEM at 37°C for 30 min. The digested tissue was passed through 45 μm strainer and the pass through was neutralized using HBSS with 0.1% BSA and 1%FBS. Following centrifugation at 400 *g* for 6 min, the cells were resuspended in red blood lysis buffer (802 mg NH_4_Cl, 84 mg NaHCO_3_, 37 mg EDTA). The cell suspension was stirred for 5 min and the lysis reaction was neutralized using DMEM. The lysed cells were then centrifuged at 400 g for 12 min and resuspended in PBS for fluorescent staining.

#### Macrophage polarization

BMM were primed using recombinant murine IFNγ (50 ng/mL) (575,302, BioLegend) for 4 h followed by overnight M1 polarization using LPS (100 ng/mL) (L2630, Sigma-Aldrich), recombinant human HMGB1 (rHMGB1) (500 ng/mL) (1690-HMB, R&D), HFHG (500 μM PA in 0.5% BSA and 6 g/L D-glucose), HFHG + rHMGB1. For M2 polarization, the macrophages were stimulated with recombinant murine IL4 (A21323, Antibodies.com) (20 ng/mL) for 24 hs. The macrophages were also stimulated using H9C2 conditioned media for 24 hs. The conditioned medium was obtained following CHOP overexpression, HFHG stimulation in PAK2-knockdown and overexpressing, and Glycyrrhizin (8 h) treated and HFHG (12 h) stimulated cells.

#### Flow cytometry staining

Immunofluorescence was performed on single-cell suspensions from heart digests and BMM. 1-4 x10^6^ cells in a final volume of 100 μL were blocked with anti-mouse CD16/32 (1:100 in PBS) (101319, BioLegend) for 10 mins on ice. The cells were then incubated with conjugated anti-mouse antibodies of interest (Key resources table) diluted in flow cytometry staining buffer (eBioscience) for 30 mins at room temperature in the dark. The cells were washed three times with flow cytometry staining buffer. The single-cell suspension was also stained with DAPI (2.5 μg/mL in PBS) and incubated for 15 mins at room temperature, protected from light. Following washing, the pellets were resuspended in 100 μL PBS. The samples were run on BD Biosciences LSRFortessa running BDFACS software. A channel for auto-fluorescence (488 529/24) (FITC) was added to the acquisition panel. A single compensation matrix was generated from single antibody controls. The data was acquired using corresponding channels: CD86: 640 (780/60), CD206: 640 (670/30), Ly6G: 640 (730/45), CD45: 405 (525/50), F4/80: 488 (586/15) and DAPI: 405 (450/50). Acquired data was analyzed using FlowJo v10 software. Dead cells (DAPI+), and any Ly6G + leukocytes were excluded from analysis of macrophages. Derive parameter function was used to calculate M1/M2 from fluorescence intensity from corresponding channels (M1 = 640 780/60; M2 = 640 670/30).

### Quantification and statistical analysis

#### Histological imaging and analysis

Masson’s, H&E-stained and DAB-developed sample images were acquired on a 3D-Histech Pannoramic-250 microscope slide-scanner using a Zeiss 20x/0.80 Plan Apochromat objective. Snapshots of the slide-scans were taken using the Case Viewer software (3D-Histech) with whole-heart images for Masson’s and section images at ×40 magnification for H&E and DAB developed samples. Heart images were analyzed using color thresholding for Masson’s, and cross-sectional area measurement (μm^2^) for H&E on ImageJ software. Average cross-sectional area of 100 cardiomyocytes was measured for each sample. To determine the nuclear H-score, 10 images per heart were analyzed using IHC profiler plugin (Varghese et al., 2014). The nuclear pixel intensity was assigned a score as high positive (3+), positive (2+), low positive (1+) and negative (0).

Fluorescent-labelled slides were imaged using a Zeiss Axioimager.D2 upright microscope using a 40x/0.2 Plan-neofluar objective and captured using a Coolsnap HQ2 camera (Photometrics) through Micromanager software v1.4.23. Specific band pass filter sets for DAPI (nuclei), FITC and Texas Red were used to prevent bleeding through from one channel to the next. Images were then processed and analyzed using Image J software. For 3D reconstruction, images were collected on a Leica TCS SP8 AOBS inverted confocal using a HC PL APO CS2 40x/1.30 OIL objective and 0.75 confocal zoom. The confocal settings were as follows, pinhole 1 airy unit, scan speed 400 Hz unidirectional, format 1024 × 1024. Images were collected using hybrid detectors with the following detection mirror settings; DAPI 415–472 nm; AlexaFlour488 497–572 nm; AlexaFlour594 604–752 nm using the white light laser with 405 nm (20%), 488 nm (16%) and 594 nm (12%) laser lines, respectively. The images were collected sequentially and for acquiring 3D optical stacks the confocal software was used to determine the optimal number of Z sections. The 3D images were reconstructed using Imaris Microscopy Image Analysis software (Bitplane AG, Switzerland).

For immunocytofluorescence analysis, percentage CHOP total pixel intensity in nuclei area was quantified by Image J macro: Intensity Ratio Nuclei Cytoplasm Tool (RRID:SCR_018573).

#### Data analysis

Data are presented as bar or dot plots showing mean ± SEM. The animal sample size (*N*) required to detect 5.20 difference of IVRT mean with SD of 2.58 between mice fed with chow or HFHSD was determined based on a power analysis (statistical power ≥0.8 and α < 0.05), suggesting a minimum of *N* = 4 per group. In figure legends, indicated *N* and n represent biological and technical replicates, respectively. Data were analyzed using one-way or two-way ANOVA followed by post hoc tests where appropriate. Comparisons between two groups were performed using Student’s *t* test. For data with skewed distribution, non-parametric tests were used. Correlations were determined using Pearson’s coefficient (R). Statistical analysis was performed using the GraphPad Prism 9 software and *p* values < 0.05 were considered statistically significant.

## Data Availability

Raw data from Figure 1, Figure 2, Figure 3, Figure 4, Figure 5, Figure 6, Figure 7 and Figure 1, Figure 2, Figure 3, Figure 4, Figure 5, Figure 6, Figure 7 were deposited on Mendeley at https://doi.org/10.17632/b5y5kvjdtt.1. Any additional information required to reanalyse the data reported in this paper is available from the lead contact upon request.
