# Supplementary material for: Paracrine signal emanating from stressed cardiomyocytes aggravates inflammatory microenvironment in diabetic cardiomyopathy
Source: iScience. 2022 Feb 23;25(3):103973. doi: 10.1016/j.isci.2022.103973 (PMC8905320; doi:10.1016/j.isci.2022.103973)
Supplement: Document S1. Figures S1–S13 and Tables S1–S6 [file mmc1.pdf]

iScience, Volume *25*

Supplemental information

Paracrine signal emanating from stressed cardiomyocytes aggravates inflammatory microenvironment in diabetic cardiomyopathy

Namrita Kaur, Andrea Ruiz-Velasco, Rida Raja, Gareth Howell, Jessica M. Miller, Riham R.E. Abouleisa, Qinghui Ou, Kimberly Mace, Susanne S. Hille, Norbert Frey, Pablo Binder, Craig P. Smith, Helene Fachim, Handrean Soran, Eileithyia Swanton, Tamer M.A. Mohamed, Oliver J. Müller, Xin Wang, Jonathan Chernoff, Elizabeth J. Cartwright, and Wei Liu

Table S1: Electrocardiogram parameters of HFHSD and chow-fed mice, related to Figure 1. Data presented as mean ± SEM. *HR: heart rate, bpm: beats per minute, QT(c): corrected QT.* p values determined by ANOVA followed by post-hoc test.

		HFHSD			
Weeks	Chow	8	12	16	24
HR (bpm)	773.4±12.82	750.5±12.02	777.9±7.89	744.8±3.31	730.3±9.35 $^{p=0.02}$
RR (ms)	77.65±1.29	80.37±1.43	77.17±0.77	80.56±0.36	82.31±1.06 $^{p=0.02}$
QRS (ms)	11.85±0.41	11.14±0.53	8.92±0.34	11.56±0.45	11.66±0.74
QT(c) (ms)	76.26±1.53	74.14±4.36	72.08±2.60	74.31±4.28	75.08±1.91
P duration (ms)	11.35±1.01	9.90±0.61	9.98±0.57	11.98±1.19	11.68±0.22
JT (ms)	9.40±0.21	9.80±1.15	11.05±0.57	9.49±0.81	9.83±0.32

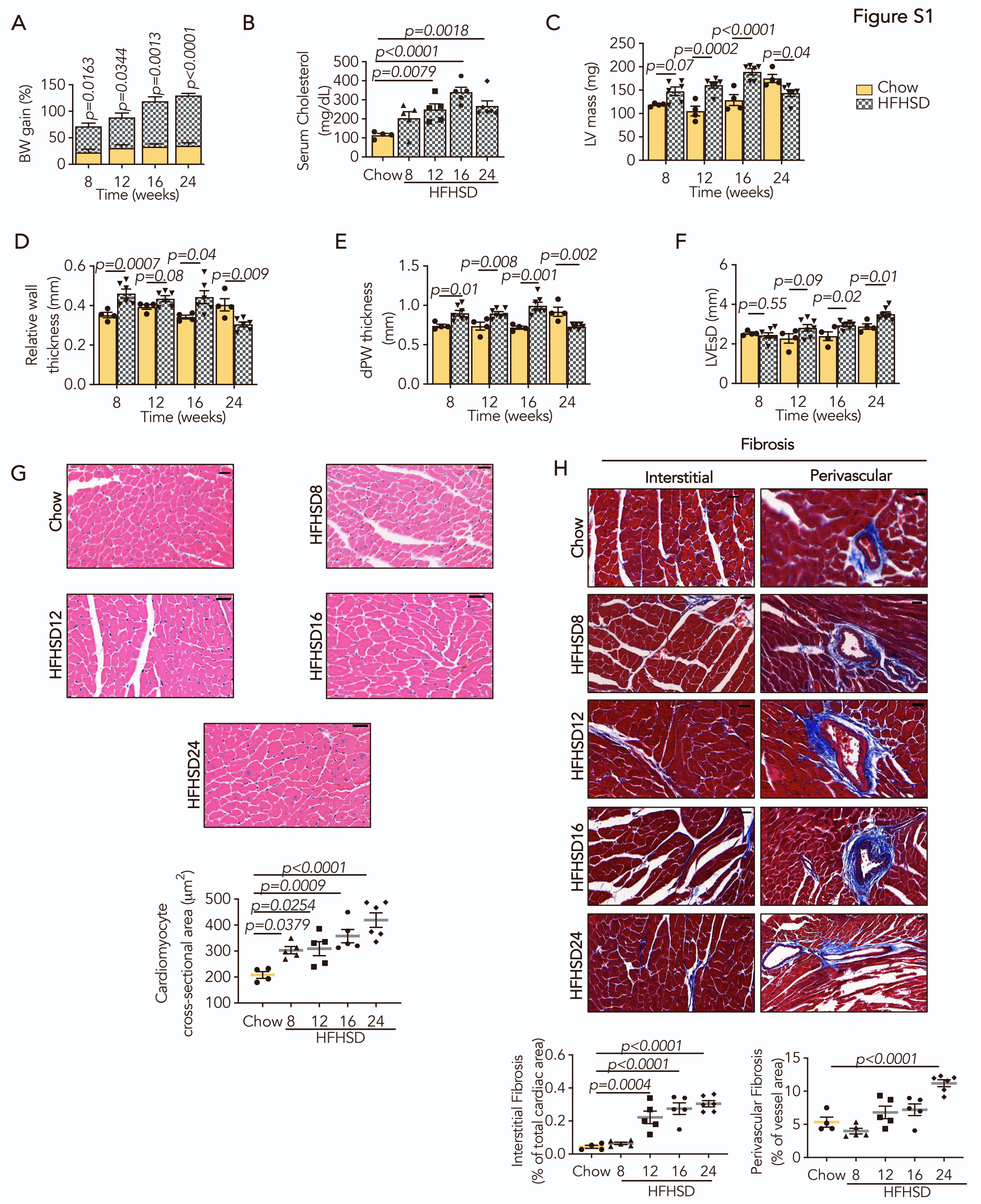

Figure S1: BW, serum cholesterol and cardiac morphology from C57BL/6J mouse in time-course studies, related to Figure 1: Data graphs showing **A** BW gain and **B** serum cholesterol (N=4-5/group). **C** Left Ventricular (LV) mass, **D** relative wall thickness, **E** diastolic posterior wall thickness (dPW) and **F** LV-end systolic diameter (LVEsD) showing ventricular remodeling (N=6/group). **G** Cardiomyocyte size measured from H&E-stained heart sections (N=4-8, scale bar: 20μm). **H** Interstitial (scale bar: 20μm) and perivascular (scale bar: 50μm) fibrosis from Masson's trichrome staining (N=4-6/group). All data are presented as mean ± SEM. *p* values (shown in each panel) versus corresponding controls determined by by ANOVA with Tukey's post hoc tests.

Figure S2

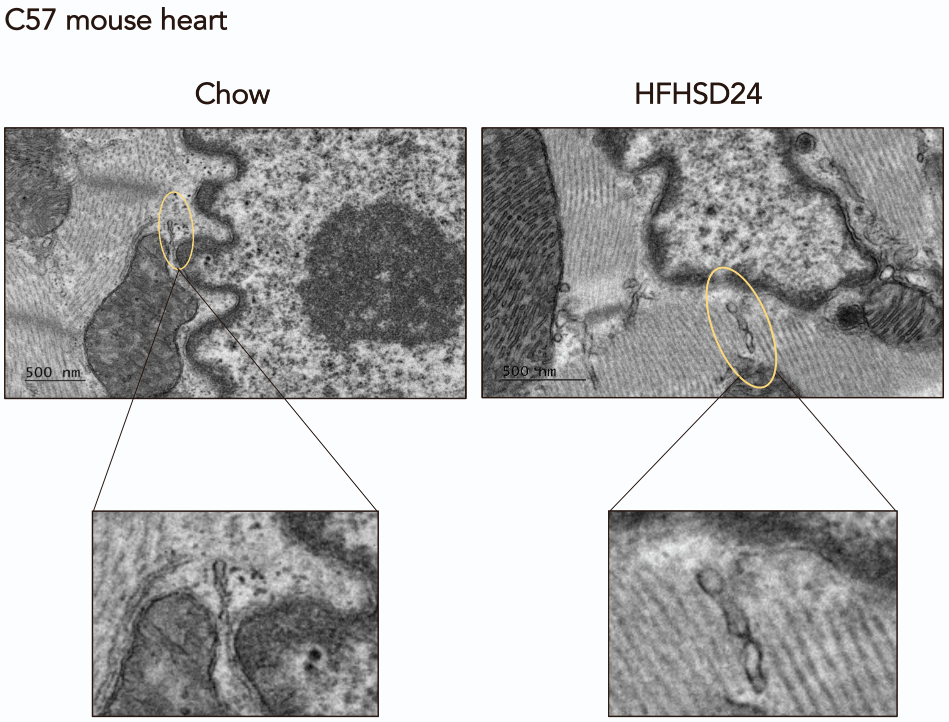

Figure S2: Comparison of ER structural integrity, related to Figure 1: Representative transmission electron microscope images (TEM) from N=4/group displaying swollen ER (encircled) following HFHSD in C57BL/6J hearts.

C57 mouse heart
Ly6G immunohistochemistry

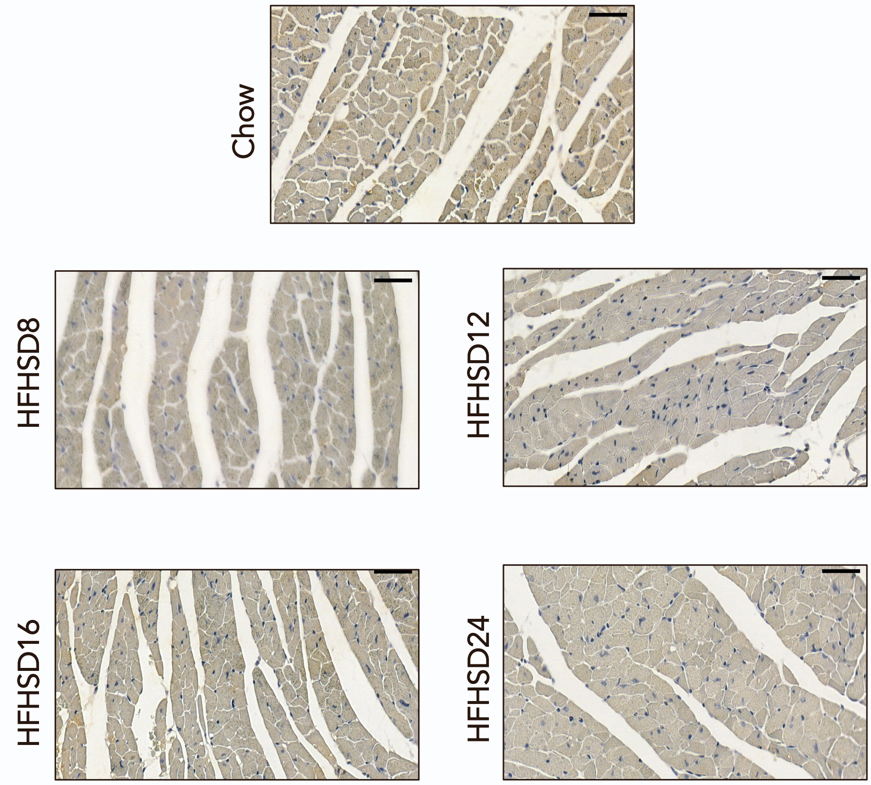

Figure S3: Neutrophil infiltration in heart sections, related to Figure 2: Representative immunohistochemical images for Ly6G from C57BL/6J hearts (scale bar: 40μm).

Figure S4

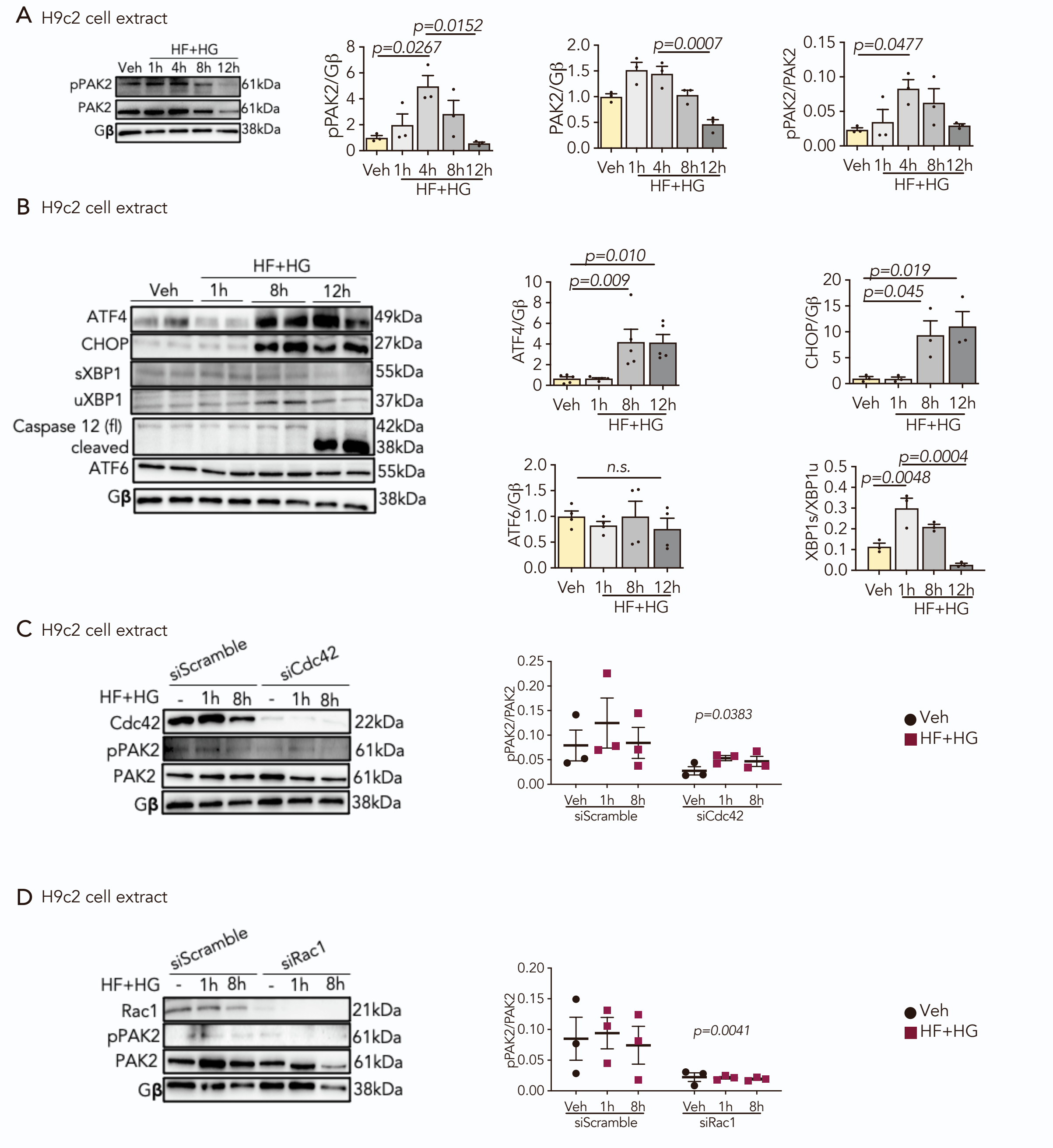

Figure S4, PAK2 and UPR activation following HFHG, related to Figure 3: Immunoblot and quantification (normalized to vehicle (Veh), AU) of **A** pPAK2 and PAK2 expression; **B** ER stress markers including ATF4, CHOP, spliced XBP1 (XBP1s), unspliced XBP1 (XBP1u), full length (fl) and cleaved caspase 12 and ATF6 in high fatty acid (500µM) and high glucose (33mM) (HF+HG) stimulated H9C2s; Immunoblot images and quantification of PAK2 activation in H9C2 stimulated with HF+HG with **C** Cdc42 knockdown using siCdc42 and **D** Rac1 knockdown using siRac2, compared to siScramble. N=3-6 experiments represented by data points. All data are presented as mean ± SEM. *p* values (shown in each panel) versus corresponding controls by ANOVA with Tukey's/Sidak's post hoc tests.

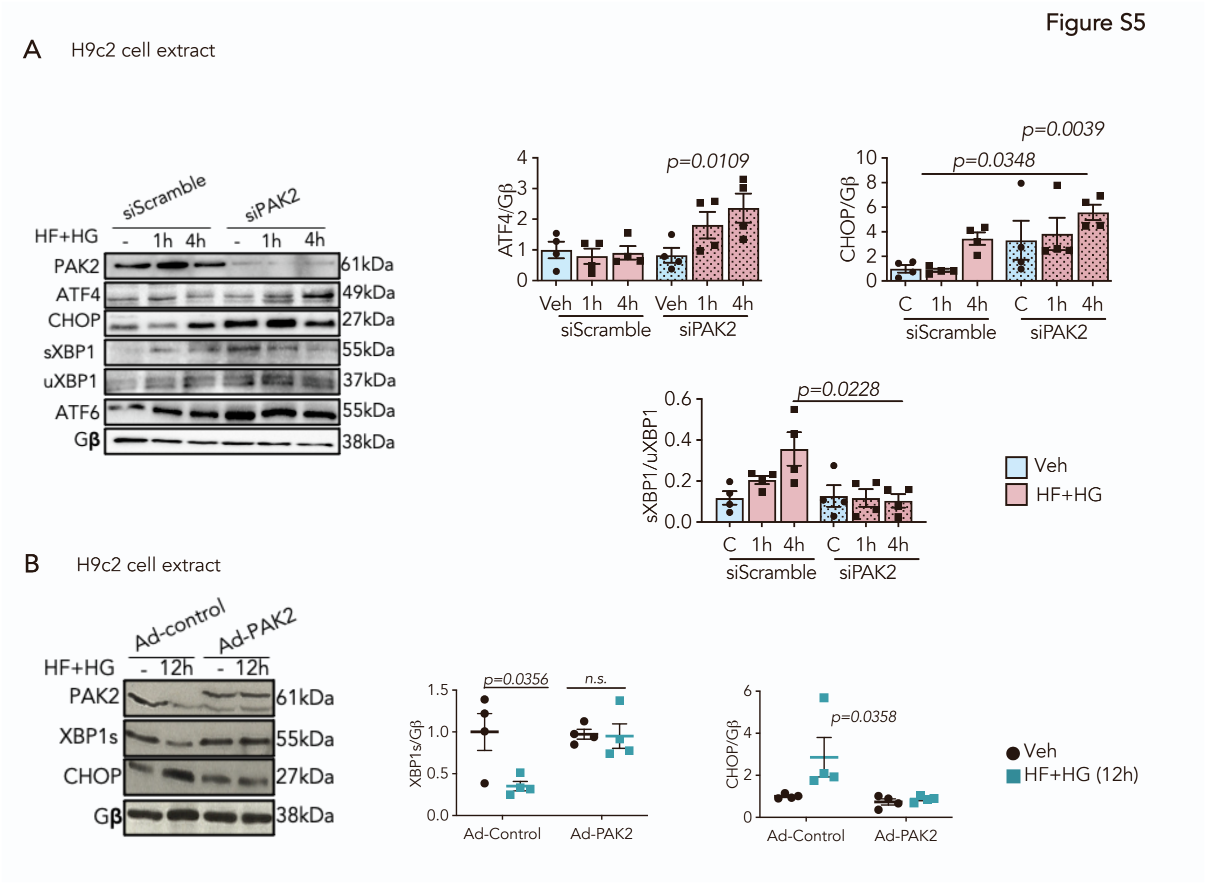

Figure S5: ER stress following either Pak2 knockdown or overexpression, related to Figure 3: Immunoblot analyses of ER stress markers depicting opposing homeostasis following HF+HG stimulation in H9C2s with **A** Pak2 knockdown using siPAK2, or with **B** Pak2 overexpression using adenovirus-PAK2 (Ad-PAK2). Gβ is loading control. Each data point represents one experiment (N=4). All data are presented as mean ± SEM. *p* values (shown in each panel) versus corresponding control determined by ANOVA with Tukey's/Sidak's post hoc tests.

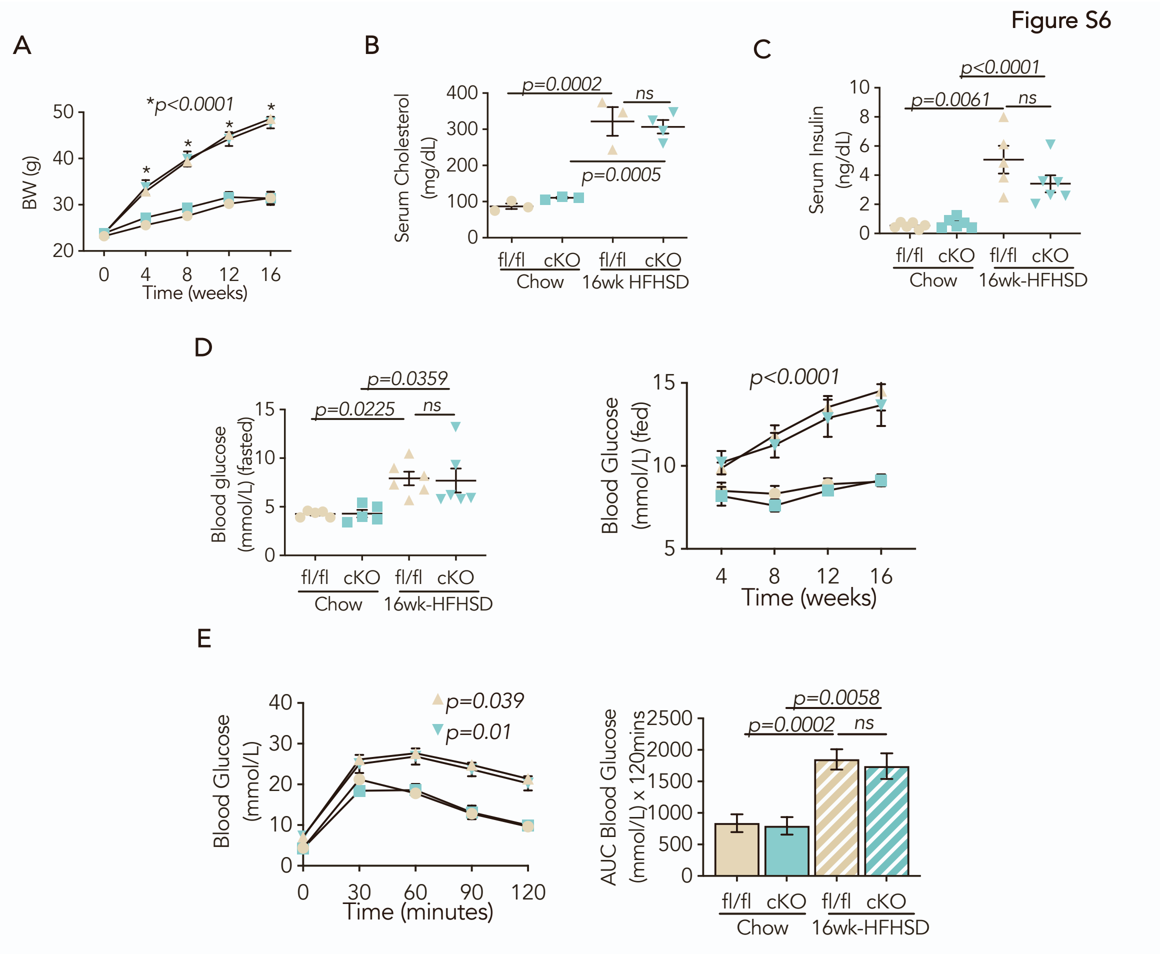

Figure S6: Systemic metabolic measurements from *Pak2*$^{fl/fl}$ *and Pak2*^{cKO} *mice*, related to Figure 3: Data graphs showing **A** BW, **B** serum cholesterol, **C** serum insulin, **D** fasted and fed blood glucose and **E** intraperitoneal glucose tolerance test after 16 wks of HFHSD feeding and bar graph displaying AUC in PAK2 mice. N=9-12/group in **A** and **E**, N=3-6/group in **B-D.** All data are presented as mean ± SEM. *p* values (shown in each panel) versus corresponding control determined by ANOVA with Tukey's/Sidak's post hoc tests.

Table S2: Echocardiography parameters of *Pak2*$^{fl/fl}$ and *Pak2*^{cKO} mice, related to Figure 3: Data presented as mean ± SEM. *Left ventricle end-systolic diameter (LVEsD), systolic posterior wall (sPW), d (diastolic) and s (systolic) volume, stroke volume (SV).* p values determined by ANOVA followed by post-hoc test.

	Chow		HFHSD	
**4-Weeks**	*Pak2*$^{fl/fl}$	*Pak2*^{cKO}	*Pak2*$^{fl/fl}$	*Pak2*^{cKO}
LVEsD (mm)	2.444±0.129	2.604±0.160	2.577±0.100	2.638±0.136
sPW (mm)	1.032±0.088	0.903±0.064	0.844±0.028	1.002±0.049
dVol (µL)	72.24±8.05	58.49±9.28	71.82±3.95	84.58±5.86
sVol (µL)	16.62±2.16	19.95±3.49	19.15±2.30	20.58±3.02
SV (µL)	55.61±6.85	45.33±5.41	52.67±3.09	64.00±3.90
**8-Weeks**	*Pak2*$^{fl/fl}$	*Pak2*^{cKO}	*Pak2*$^{fl/fl}$	*Pak2*^{cKO}
LVEsD (mm)	2.513±0.138	2.550±0.085	2.569±0.064	2.774±0.144
sPW (mm)	0.866±0.015	0.891±0.051	0.963±0.023	1.124±0.053
dVol (µL)	75.18±5.40	70.28±10.15	75.47±3.36	84.43±6.49
sVol (µL)	18.34±3.25	17.77±1.73	18.26±1.35	24.06±4.09
SV (µL)	56.83±2.95	60.84±5.37	57.21±2.49	60.37±4.68
**12-Weeks**	*Pak2*$^{fl/fl}$	*Pak2*^{cKO}	*Pak2*$^{fl/fl}$	*Pak2*^{cKO}
LVEsD (mm)	2.365±0.097	2.493±0.135	2.451±0.084	2.937±0.145$^{p=0.049}$
sPW (mm)	0.893±0.023	0.910±0.029	1.095±0.036	1.096±0.076
dVol (µL)	77.60±4.05	79.03±11.21	74.88±4.32	98.29±6.66$^{p=0.04}$
sVol (µL)	14.74±1.99	17.55±3.06	16.23±1.68	28.26±3.87$^{p=0.005}$
SV (µL)	62.85±2.66	69.04±7.06	58.64±3.73	70.03±4.87
**16-Weeks**	*Pak2*$^{fl/fl}$	*Pak2*^{cKO}	*Pak2*$^{fl/fl}$	*Pak2*^{cKO}
LVEsD (mm)	2.559±0.106	2.739±0.076	2.659±0.084	3.429±0.142$^{p=0.0015}$
sPW (mm)	1.014±0.074	0.980±0.032	1.153±0.031	0.912±0.026$^{p<0.001}$
dVol (µL)	88.26±4.85	91.43±9.54	84.62±4.72	116.9±9.91$^{p=0.05}$
sVol (µL)	18.71±2.20	21.97±1.88	20.54±1.82	44.22±5.81$^{p=0.01}$
SV (µL)	69.55±3.71	78.14±4.19	64.08±3.41	72.64±5.67

Table S3: Electrocardiogram parameters of *Pak2*$^{fl/fl}$ and *Pak2*^{cKO} mice, related to Figure 3: Data presented as mean ± SEM. *HR: heart rate, bpm: beats per minute, QT(c): corrected QT*. p values determined by ANOVA followed by post-hoc test.

	Chow		HFHSD	
**4-Weeks**	*Pak2*$^{fl/fl}$	*Pak2*^{cKO}	*Pak2*$^{fl/fl}$	*Pak2*^{cKO}
HR (bpm)	723.2±13.07	643.5±48.18	733.7±9.26	694.6±14.28
RR (ms)	83.15±1.53	95.26±8.26	81.87±1.03	86.71±1.91
QRS (ms)	10.54±0.71	11.25±0.67	11.00±0.36	13.02±0.47$^{p=0.03}$
QT(c) (ms)	62.27±2.86	69.08±3.32	74.55±5.23	76.97±3.48
P duration (ms)	8.78±0.95	6.83±1.57	10.44±0.91	9.01±0.58
**8-Weeks**	*Pak2*$^{fl/fl}$	*Pak2*^{cKO}	*Pak2*$^{fl/fl}$	*Pak2*^{cKO}
HR (bpm)	666.0±14.89	651.4±30.46	722.2±18.70	725.1±8.34
RR (ms)	90.60±2.07	93.41±4.76	83.53±2.43	82.92±0.95
QRS (ms)	11.88±0.60	13.30±0.59	10.93±0.36	12.91±0.22$^{p=0.004}$
QT(c) (ms)	63.99±2.34	75.42±3.86	74.82±2.42	91.39±3.67
P duration (ms)	9.88±0.80	9.15±0.86	10.16±0.83	19.16±0.87
**12-Weeks**	*Pak2*$^{fl/fl}$	*Pak2*^{cKO}	*Pak2*$^{fl/fl}$	*Pak2*^{cKO}
HR (bpm)	703.5±20.37	690.0±22.9	721.8±18.70	694.3±8.34
RR (ms)	85.74±2.58	87.49±2.97	83.35±1.72	87.01±1.99
QRS (ms)	11.21±0.45	14.35±0.69	11.27±0.36	13.45±0.69
QT(c) (ms)	63.30±2.01	84.51±3.86	69.79±2.42	88.93±3.67$^{p=0.008}$
P duration (ms)	10.44±0.32	11.00±1.37	10.5±0.86	11.29±0.33
**16-Weeks**	*Pak2*$^{fl/fl}$	*Pak2*^{cKO}	*Pak2*$^{fl/fl}$	*Pak2*^{cKO}
HR (bpm)	702.8±21.19	708.9±15.68	706.9±4.75	702.7±13.81
RR (ms)	85.79±2.82	85.41±2.34	85.12±0.64	85.57±1.79
QRS (ms)	11.25±0.52	14.95±0.76	12.09±0.47	13.9±0.63
QT(c) (ms)	64.26±2.46	82.79±4.79	70.25±3.03	96.73±7.04$^{p=0.04}$
P duration (ms)	10.78±0.49	9.42±0.86	11.11±0.66	10.77±0.87

Figure S7

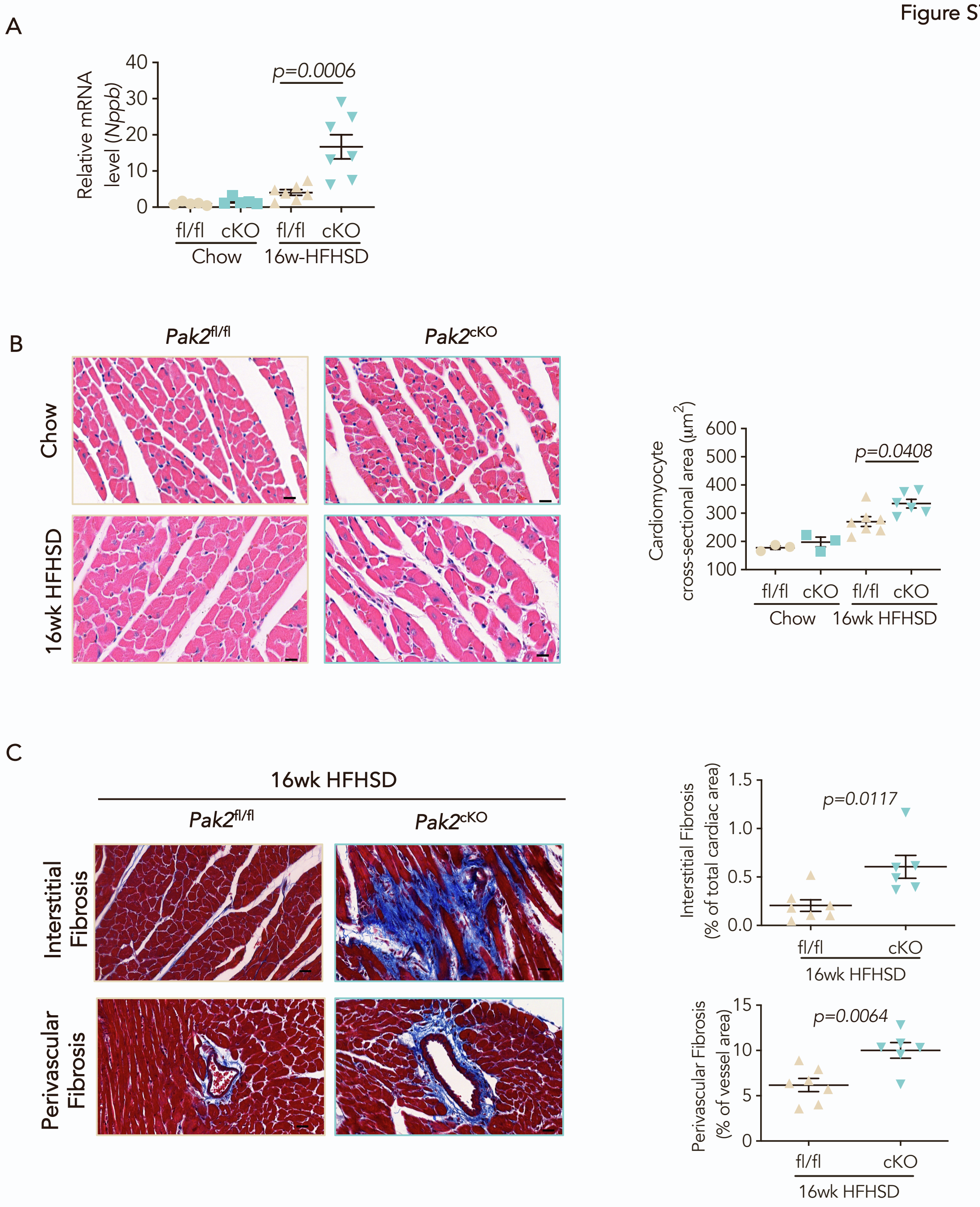

Figure S7: *Nppb* level and heart morphology from *Pak2$^{fl/fl}$* and *Pak2^{cKO}* mice related to Figure 3: Data graphs showing **A** Relative mRNA expression of *Nppb* (chow normalized to 1, AU*)* (N=6/group) in PAK2 mice**. B** Cardiomyocyte size measured from H&E-stained heart sections (N=3-6, scale bar: 2mm). **C** Interstitial and perivascular fibrosis from Masson's trichrome staining (N=6-7/group, scale bar: 2mm). All data are presented as mean ± SEM. *p* values (shown in each panel) determined by two-tailed Student's t-test in **C,** and by ANOVA with Tukey's post hoc test in **A-B**.

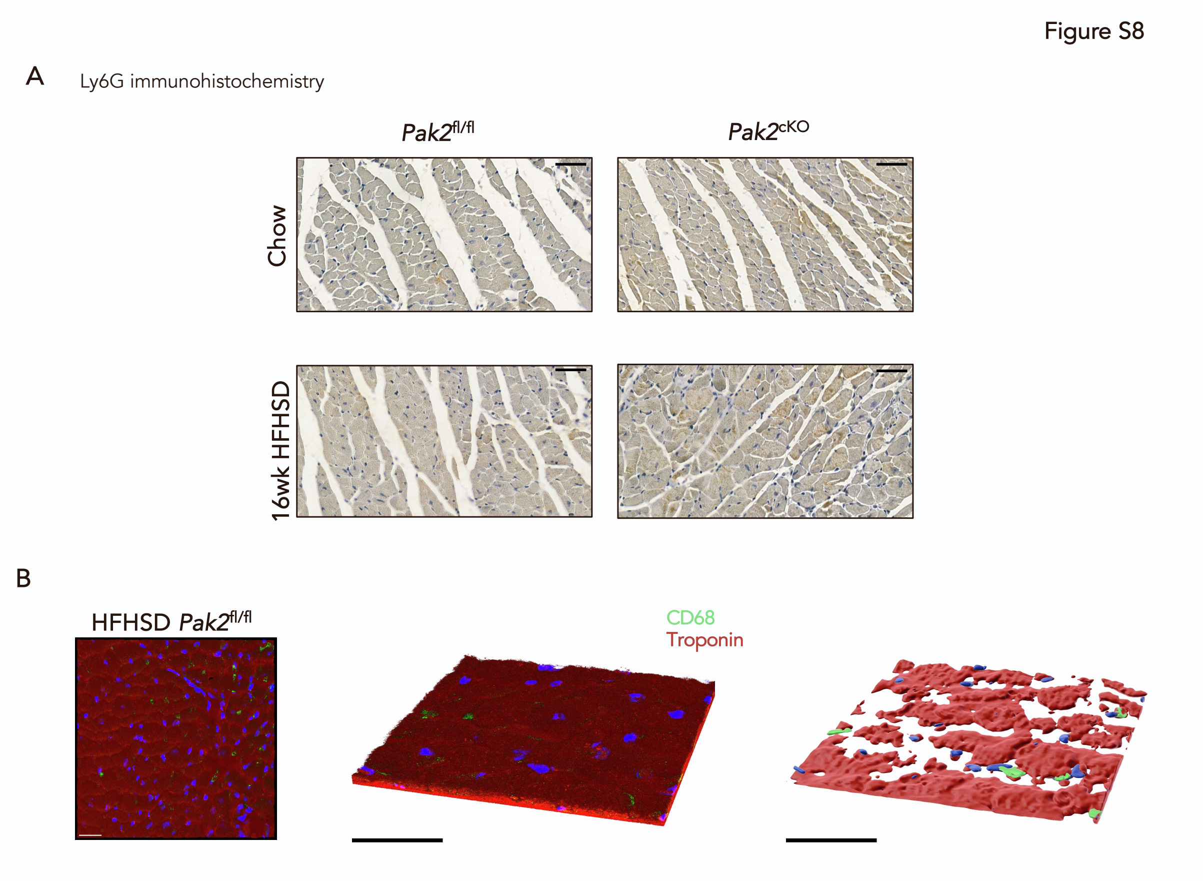

Figure S8: Immune cell (neutrophils and macrophages) staining from *Pak2$^{fl/fl}$* *and Pak2^{cKO}* *mice* related to Figure 3: Representative immunohistochemical images for Ly6G from **A** *Pak2$^{fl/fl}$* and *Pak2^{cKO}* hearts (scale bar: 40µm). **B** Representative images of CD68 macrophages (green) and cardiomyocytes (red) stained with troponin from *Pak2$^{fl/fl}$* heart section, displaying zoomed blend mode and 3D-surface reconstruction from left to right (scale bar: 50mm).

Figure S9

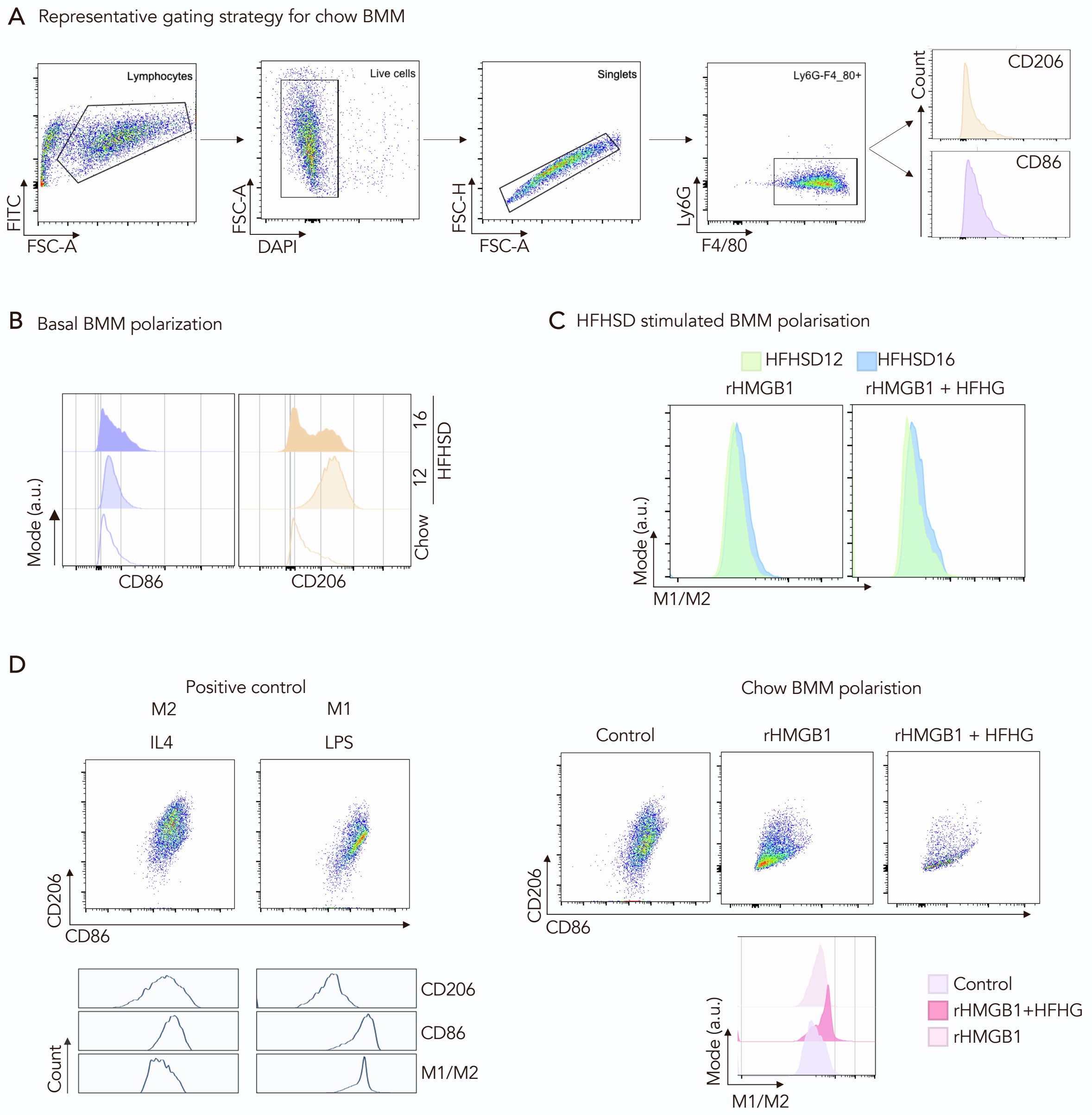

Figure S9: Gating strategy and representative flow cytometry images, related to Figure 4: A Following gating using granularity and size (FSC and SSC), macrophages were selected as Ly6G$^-$ F4/80$^+$ (parent population) from BM-derived macrophages. Histograms normalised to mode displaying **B** CD206$^+$ and CD86$^+$ macrophages (parent population) from chow and HFHSD-fed mice, and **C** M1/M2 following rHMGB1 stimulation with and without HFHG. M1/M2 from HFHSD BMM calculated using derive parameter function on FlowJo from corresponding fluorescent channels of M1 (CD86) and M2 (CD206). **D** Dot plots representing CD206 and CD86 macrophages gated from Ly6G$^-$ F4/80$^+$ cells following IL4 (20ng/mL), lipopolysaccharide (LPS) (100ng/mL), control, rHMGB1 (500ng/mL) with and without HFHG stimulation from chow-fed mice. Corresponding histograms (normalised to mode, AU) for CD206, CD86 and M1/M2 shown below. Total events acquired/experiment= 10000. FITC is marker for auto-fluorescence. Graphs are representative of data presented in Figure 4.

Figure S10

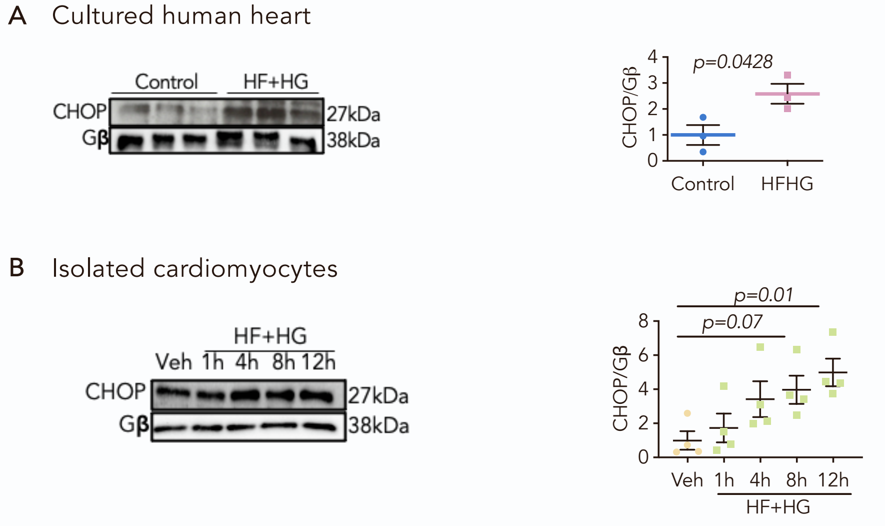

Figure S10: CHOP expression in either cultured human heart slice or rat primary cardiomyocytes, related to Figure 5: Immunoprecipitation images and quantification from **A** cultured human heart tissue (n=3, technical repeat), and **B** isolated rat cardiomyocytes (N=4), stimulated with HF+HG displaying maladaptive ER stress response via CHOP. Gβ is loading control. All data are presented as mean ± SEM. *p* values (shown in each panel) determined by two-tailed Student's t-test in **A** and by ANOVA with Tukey's post hoc test in **B**.

Figure S11

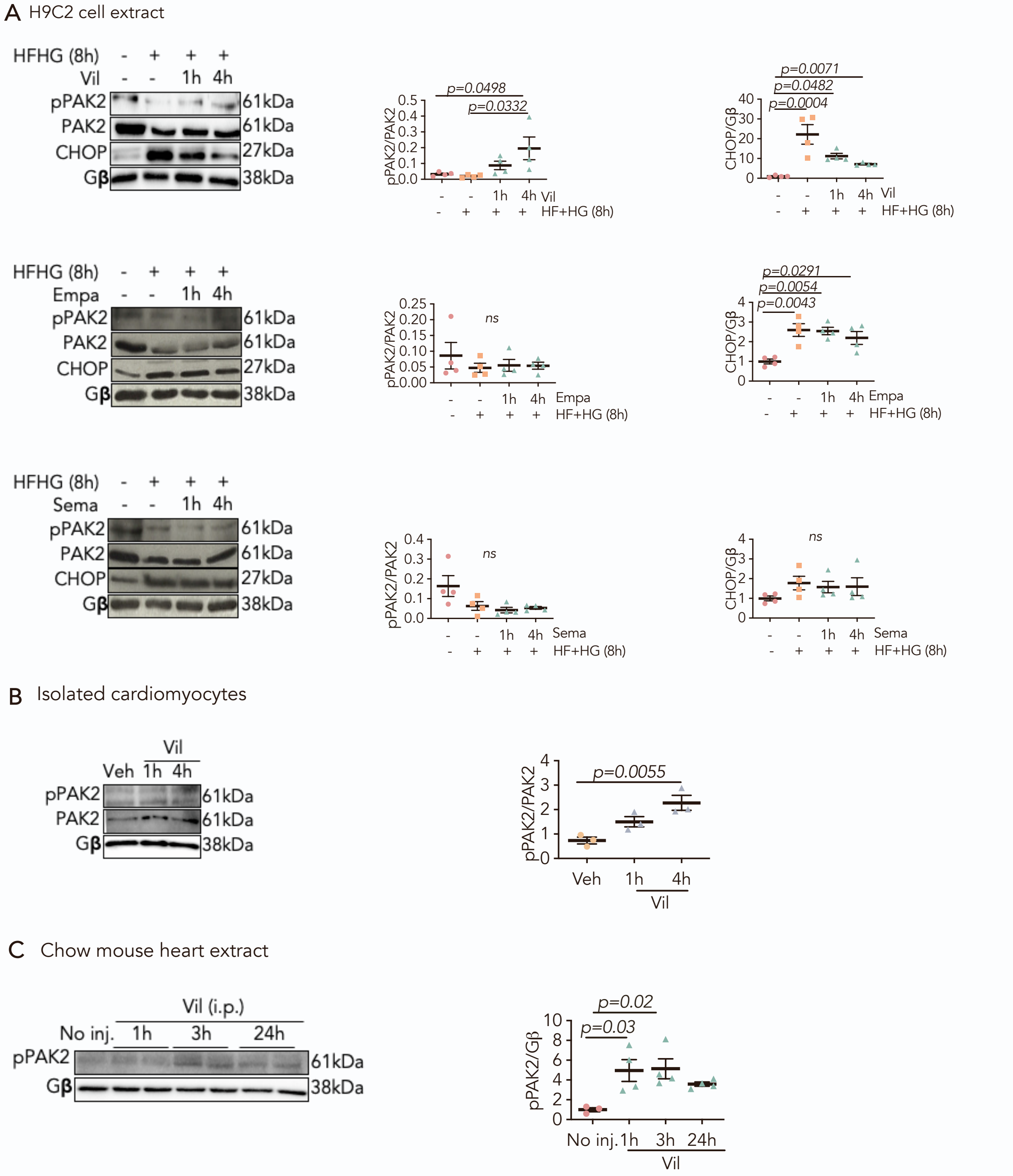

Figure S11: PAK2 activation following drug administration, related to Figure 6: A Representatives immunoblot images and quantification from H9c2 with HFHG followed by Vildagliptin, Semaglutide (Sema) and Empagliflozin (Empa) stimulation (N=4/cohort). **B** Representatives immunoblot images and quantification of PAK2 activation from isolated adult rat cardiomyocytes in the fact of Vildagliptin (Vil) (20µM) stimulation (N=3). **C** Representatives immunoblot images from hearts of 6-wk-old C57BL/6J mice displaying PAK2 activation in the myocardium following intraperitoneal administration of Vildagliptin (5mg/kg) (N=2 mice/group). Quantification from n=2 (technical repeat) western blots. Gβ is loading control. Data are presented as mean ± SEM. *p* values determined by ANOVA with Tukey's post hoc test.

Table S4: Echocardiography parameters of *Pak2*^{cKO} mice following treatment, related to Figure 6*:* Data presented as mean ± SEM. p values determined by ANOVA followed by post-hoc test. P to chow; * to Vil; # to HFHSD only.

	Chow	HFHSD		
**12-wks**			Vil	Vil + AAV9-*Pak2*
FS (%)	40.3±2.4	35.8±3.3	32.2±2.0$^{p=0.04}$	40.4±2.3$^{*p=0.04}$
EF(%)	81.8±1.4	76.1±3.4	68.4±2.7$^{p=0.006}$	78.2±2.7$^{*p\text{-}0.05}$
RWT	0.30±0.02	0.40±0.04$^{p=0.0021}$	0.40±0.02$^{p=0.0042}$	0.38±0.01$^{p=0.0173}$
IVRT	10.7±0.56	15.2±0.83$^{p=0.0004}$	15.7±0.49$^{p<0.0001}$	12.8±0.79$^{*p=0.0375}$
E/A	1.49±0.07	1.51±0.16	1.90±0.13	1.57±0.05
LV mass	127.7±12.8	157.6±11.3	136.9±10.1	114.0±9.2$^{\#p=0.03}$
LV mass/BW	3.87±0.11	3.47±0.23	3.18±0.16	$^{p=0.0008}$ 2.60±0.19$^{\#p=0.0296}$
LVEsD (mm)	2.51±0.17	2.74±0.17	2.88±0.14	2.42±0.16
LVEdD (mm)	4.35±0.18	4.44±0.15	4.24±0.14	4.03±0.12
dPW (mm)	0.65±0.03	0.89±0.06$^{p<0.0001}$	0.84±0.02$^{p<0.0016}$	0.77±0.03
sPW (mm)	0.95±0.03	1.20±0.10$^{p<0.0012}$	1.04±0.02	0.927±0.026$^{\#p=0.004}$
dVol (µL)	88.17±6.42	93.43±9.71	81.01±8.07	69.59±6.02
sVol (µL)	15.91±1.57	22.70±3.93	25.86±3.83	15.75±3.19
SV (µL)	72.26±7.20	70.73±7.87	55.16±5.17	53.84±3.56
**16-Wks**			Vil	Vil + AAV9-*Pak2*
EF(%)	80.8±1.4	62.6±3.5$^{p<0.0001}$	58.2±2.9$^{p<0.0001}$	$^{*p=0.0002}$ 75.4±1.4$^{\#p=0.0060}$
RWT	0.31±0.01	0.30±0.02	0.31±0.01	0.34±0.01
E/A	1.66±0.40	2.07±0.09	1.82±0.22	1.62±0.07$^{\#p=0.0479}$
LV mass	135.9±9.6	157.5±16.0	109.4±6.8$^{p=0.02}$	122.5±8.4
LV mass/BW	4.21±0.29	3.25±0.32$^{p=0.0141}$	$^{p<0.0001}$ 2.38±0.11$^{\#p=0.0307}$	2.67±0.19$^{p<0.0001}$
HW/BW	4.04±0.20	3.22±0.20$^{p=0.0182}$	2.92±0.09$^{p=0.0012}$	2.91±0.19$^{p=0.0010}$
HW/TL	5.86±0.37	7.52±0.54$^{p=0.0261}$	6.36±0.22	6.43±0.31
LVEsD (mm)	2.63±0.17	3.46±0.22$^{p=0.0012}$	3.18±0.08$^{p=0.0474}$	$^{\#p=0.0009}$ 2.62±0.05$^{*p=0.0398}$
LVEdD (mm)	4.52±0.09	4.82±0.20	4.27±0.09$^{\#p=0.0351}$	4.19±0.03$^{p=0.0120}$
dPW (mm)	0.68±0.02	0.72±0.04	0.65±0.02	0.72±0.02
sPW (mm)	0.93±0.04	0.84±0.02	0.86±0.03	0.94±0.04
dVol (µL)	96.83±5.29	120.60±16.33	82.30±5.50$^{\#p=0.0129}$	77.16±1.601$^{\#p=0.004}$
sVol (µL)	19.74±1.75	40.06±9.07$^{p=0.003}$	34.11±2.49	18.93±1.10$^{\#p=0.002}$
SV (µL)	77.09±5.52	74.54±9.07	$^{p=0.0079}$ 48.18±4.45$^{\#p=0.0176}$	58.24±1.74

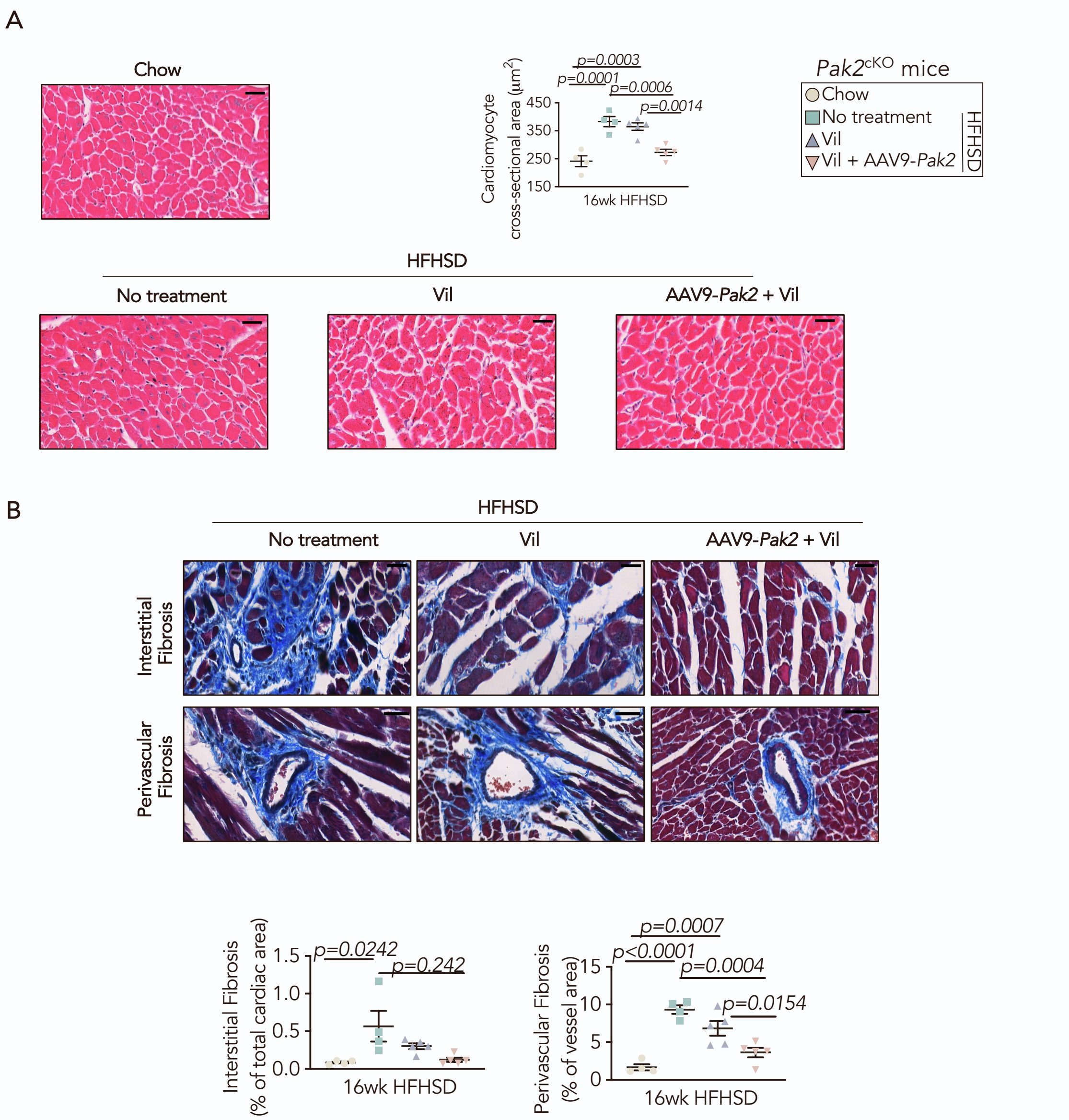

Figure S12: Cardiac morphology of *Pak2*^{cKO} mice following treatment related to Figure 6: Data graphs showing **A** cardiomyocyte size measured from H&E-stained heart sections (N=3-5/group, scale bar: 30µm). **B** Interstitial and perivascular fibrosis from Masson's trichrome staining (N=3-5/group, scale bar: 30µm). All data are presented as mean ± SEM. *p* values (shown in each panel) determined by ANOVA with Tukey's post hoc test.

Table S5: Echocardiography parameters of C57BL/6J mice following treatment, related to Figure 7*:* Data presented as mean ± SEM. p values determined by ANOVA followed by Student's t test.

	24wk HFHSD	
24-Weeks	-Gly	+Gly
LV mass	165.5±9.63	153.1±3.54
LV mass/BW	3.23±0.13	3.05±0.09
HW/BW	3.57±0.09	3.44±0.26
HW/TL	8.61±0.22	7.37±0.23 $^{p=0.0019}$
LVEdD (mm)	4.766±0.134	4.448±0.050 $^{p=0.0426}$
sPW (mm)	0.963±0.039	1.173±0.041 $^{p=0.0023}$
dVol (μL)	115.3±10.43	92.35±3.14 $^{p=0.0537}$
sVol (μL)	43.46±5.78	22.02±2.23 $^{p=0.0038}$
SV (μL)	71.84±6.61	70.33±3.53

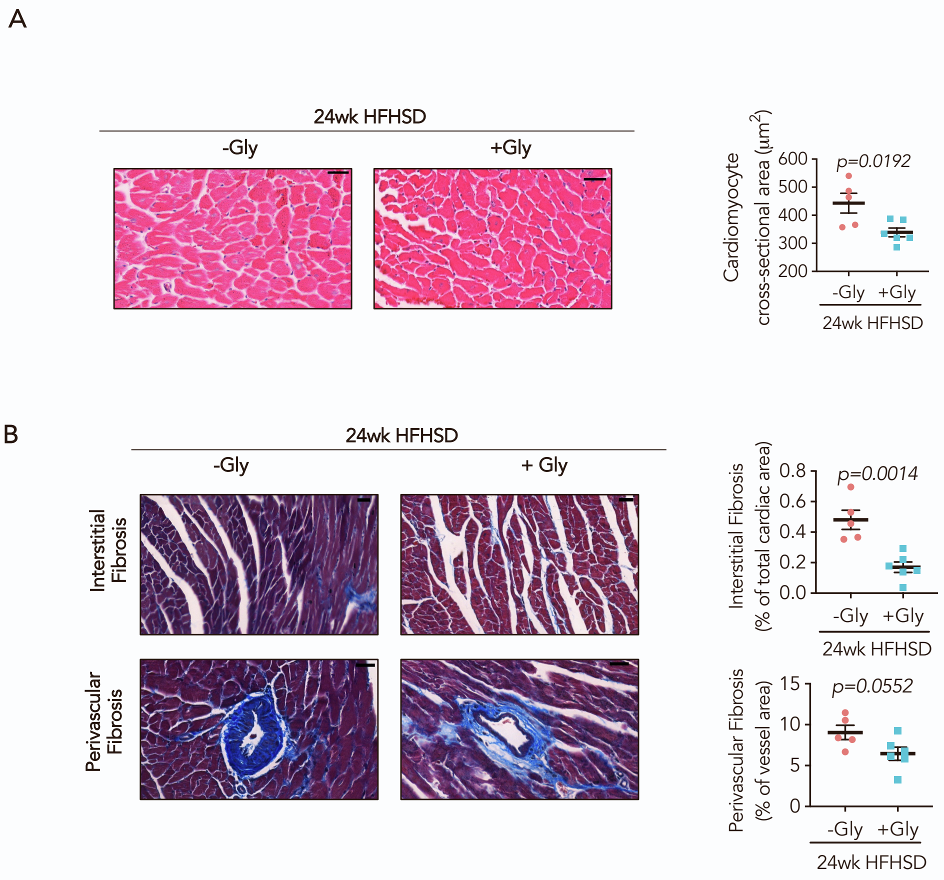

Figure S13: Cardiac morphology of C57BL/6J mice fed with HFHSD following treatment of Gly, related to Figure 7: Data graphs showing **A** cardiomyocyte size measured from H&E-stained heart sections (N=5-6/group, scale bar: 30μm). **B** Interstitial and perivascular fibrosis from Masson's trichrome staining (N=5-6/group, scale: 30μm). All data are presented as mean ± SEM. *p* values (shown in each panel) determined by two-tailed Student's t-test.

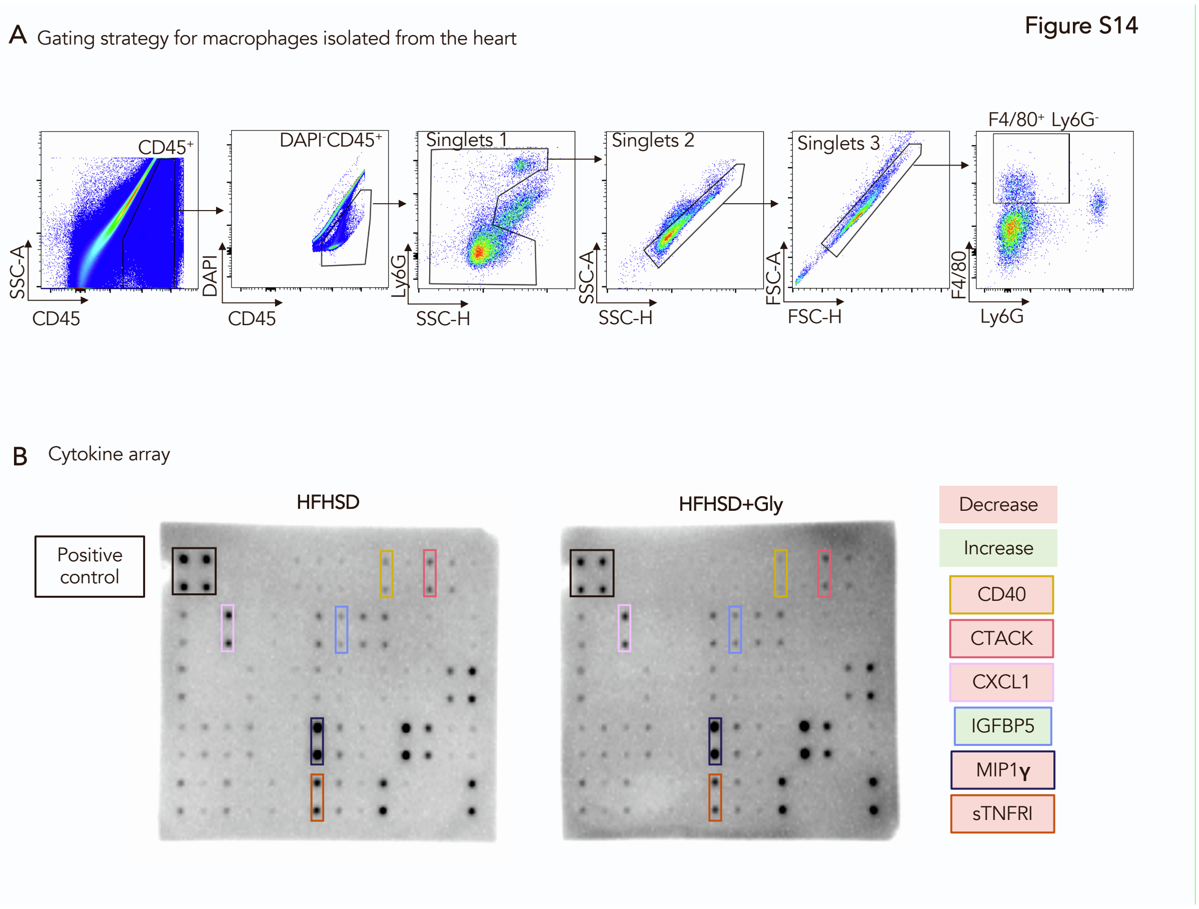

Figure S14: Gating strategy for heart flow cytometry and Cytokine array on the myocardium from C57BL/6J mice with treatment of Gly, related to Figure 7: A Following gating using granularity and size (FSC and SSC) along with CD45$^+$ cells, macrophages were selected as Ly6G$^-$ F4/80$^+$ (parent population) from single-cell suspension from the heart. All the events were collected and concatenated post-acquisition in FlowJo. **B** Membranes representing cytokine profiles of heart extracts of mice fed with HFHSD with and without glycyrrhizin (Gly). The increased (highlighted green) and decreased (highlighted red) cytokines are boxed, and corresponding names are presented. Membrane incubation was performed by pooling N=3/group.

Table S6, related to STAR Methods: qPCR primers

Target	Assay name	Cat. No.
18s	Mm_Rn18s_3_SG	QT02448075
Arg1	Mm_Arg1_1_SG	QT00134288
Ccl2	Mm_Ccl2_1_SG	QT00167832
Ccl24	Mm_Ccl24_1_SG	QT00126021
Ccl5	Mm_Ccl5_2_SG	QT01747165
Crp	Mm_Crp_1_SG	QT00255444
Gdf15	Mm_Gdf15_1_SG	QT00124481
Hmgb1	Mm_Gm6587_1_SG	QT01221409
Ifng	Mm_Ifng_1_SG	QT01038821
Il10	Mm_Il10_1_SG	QT00106169
Il11	Mm_Il11_1_SG	QT00122122
Il15	Mm_Il15_1_SG	QT00107653
Il1b	Mm_Il1b_2_SG	QT01048355
Il23a	Mm_Il23a_2_SG	QT01663613
Il6	Mm_Il6_1_SG	QT00098875
Mgl2	Mm_Mgl2_1_SG	QT00143640
Mmp9	Mm_Mmp9_1_SG	QT00108815
Mrc2	Mm_Mrc2_1_SG	QT00159621
Nos2	Mm_Nos2_1_SG	QT00100275
Retnla	Mm_Retnla_1_SG	QT00254359
Tgfb	Mm_Tgif2_1_SG	QT00298802
Tnfaip1	Mm_Tnfaip1_1_SG	QT00116564
Ym1	Mm_Chil3_1_SG	QT00108829
